# Structural basis of the specific interaction of SMRT corepressor with histone deacetylase 4

**DOI:** 10.1093/nar/gky926

**Published:** 2018-10-13

**Authors:** Suk-Youl Park, Gwang Sik Kim, Hyo-Jeong Hwang, Taek-Hyun Nam, Hee-Sae Park, Jaeyoung Song, Tae-Ho Jang, Young Chul Lee, Jeong-Sun Kim

**Affiliations:** 1 Department of Chemistry, Chonnam National University, Gwangju 61186, Republic of Korea; 2 Pohang Accelerator Laboratory, Pohang, Gyeongbuk 37673, Republic of Korea; 3 School of Biological Sciences and Technology, Chonnam National University, Gwangju 61186, Republic of Korea; 4 New Drug Development Center, Daegu-Gyeongbuk Medical Innovation Foundation, Daegu 41061, Republic of Korea

## Abstract

Modification of chromatin and related transcription factors by histone deacetylases (HDACs) is one of the major strategies for controlling gene expression in eukaryotes. The HDAC domains of class IIa HDACs repress the respective target genes by interacting with the C-terminal region of the silencing mediator for retinoid and thyroid receptor (SMRT) repression domain 3 (SRD3c). However, latent catalytic activity suggests that their roles as deacetylases in gene regulation are unclear. Here, we found that two conserved GSI-containing motifs of SRD3c are critical for HDAC4 binding. Two SMRT peptides including these motifs commonly form a β-hairpin structure in the cleft and block the catalytic entry site of HDAC4. They interact mainly with class IIa HDAC-specific residues of HDAC4 in a closed conformation. Structure-guided mutagenesis confirmed critical interactions between the SMRT peptides and HDAC4 and –5 as well as the contribution of the Arg1369 residue in the first motif for optimal binding to the two HDACs. These results indicate that SMRT binding does not activate the cryptic deacetylase activity of HDAC4 and explain how class IIa HDACs and the SMRT-HDAC3 complex are coordinated during gene regulation.

## INTRODUCTION

As the principal enzymes involved in the epigenetic control of eukaryotic transcription, histone acetyltransferases (HATs) and histone deacetylases (HDACs) play central roles in regulating chromatin remodeling via histone tail modifications (1). A failure in the balance between HAT and HDAC activities can affect the compaction level of a local chromatin region and result in improper expression of specific genes, ultimately leading to genomic instability and epigenetic diseases (2,3). Therefore, precise control of HATs and HDACs is required for regulated expression of various genes associated with signal transduction, cell growth, and cell death (3). Epigenetic studies have revealed that the therapeutic impacts of HDAC inhibitors are not limited to anticancer therapy but also affect other human diseases, including cardiovascular, neurodegenerative, and metabolic disorders (4–9).

In humans, the 18 reported HDACs can be grouped into four different classes based on their dependence on specific cofactors, similarity to yeast proteins, and phylogenetic relationships: class I (HDAC1, -2, -3 and -8), class II (HDAC4, -5, -6, -7, -9 and -10), class III (SIRTs), and class IV HDACs (HDAC11) (10). Class I, II, and IV HDACs are zinc-dependent amidohydrolases, while class III HDACs rely on nicotinamide adenine dinucleotide as a cofactor for their catalytic function. The class II enzymes are further divided into class IIa (4,5,7,9) and class IIb (6,10) according to their domain structures. Class IIa HDACs have a unique adapter domain in the N-terminal portion, which forms an extended structure and is targeted by DNA-binding transcription factors and regulatory signals, in addition to the C-terminal deacetylase domain (10). In contrast, class IIb enzymes have a characteristic long extension at the C-terminus, known as a tail domain. These two enzymes also differ in their subcellular localizations: class IIa enzymes can shuttle between the cytoplasm and nucleus in response to various regulatory signals, whereas class IIb enzymes are typically found in the cytoplasm (11–13).

Known as general corepressors, nuclear receptor-corepressor (NCoR) and SMRT form various transcriptional repression complexes that are involved in pivotal biological processes, including cell survival and differentiation during development. NCoR and SMRT are ubiquitously expressed homologous proteins and contain highly conserved autonomous repression domains (RDs), namely, RD1–RD3, in their N-terminal regions (14,15). Both SMRT and NCoR have been shown to form a large steady-state complex with HDAC3, the class I HDAC, through the association of the deacetylase-activating domain (DAD) of SMRT with HDAC3 (16,17). Class IIa HDACs, such as HDAC4 and HDAC5, were found to indirectly interact with class I HDAC3 via the SMRT/NCoR protein (17). Particularly, the C-terminal region of RD3 (RD3c) of SMRT/NCoR specifically interacts with class IIa HDACs but not with class I enzymes; therefore, SMRT/NCoR functions as a bridge factor between HDAC3 and HDAC4/-5 (18–20). In this regard, the catalytic domain of class IIa HDACs can act as a scaffold module that is responsible for recruiting the SMRT/NCoR-HDAC3 complex, regardless of its deacetylase activity (17).

According to the reported apo-structures of the catalytic domains of HDAC4 H976Y mutant (studied because of its high protein stability) and HDAC7, these enzymes have a flexible structural zinc-binding subdomain conserved only in class IIa HDACs, in addition to the catalytic zinc-containing deacetylase subdomain (21,22). Interestingly, structural analyses of inhibitor-bound HDAC4 catalytic domain (HDAC4c) revealed that the structural zinc ion-binding region can adopt two distinct conformations. The apo-structure of the H976Y mutant forms a ‘closed’ conformation that may provide a substrate path leading to the catalytic zinc ion at the active site, while inhibitor-bound structures show an ‘open’ conformation with a disordered structural zinc ion-binding subdomain (21,23). The closed conformation, rather than the open conformation, has been suggested to be a biologically relevant structure (23). Therefore, structural evaluation of the closed conformation of HDAC4c complexed with SMRT RD3c (SRD3c) may provide a better rationale to design biologically active HDAC4 inhibitors, which are distinguished from known chemical inhibitors specific for class IIa HDACs (23).

In our previous report, we found that the rims of the catalytic entry sites of HDAC4/-5 were the major binding surfaces of the SMRT corepressor (20). Here, we revealed that two conserved GSI-containing motifs of SRD3c are critical for HDAC4 binding. Structural studies show that two peptides derived from these SRD3c motifs interact in the cleft of the catalytic entry site with the closed form of HDAC4c. The detailed interactions between the SMRT peptides and HDAC4c and HDAC5c have been confirmed.

## MATERIALS AND METHODS

### Plasmids

**Figure 1.**
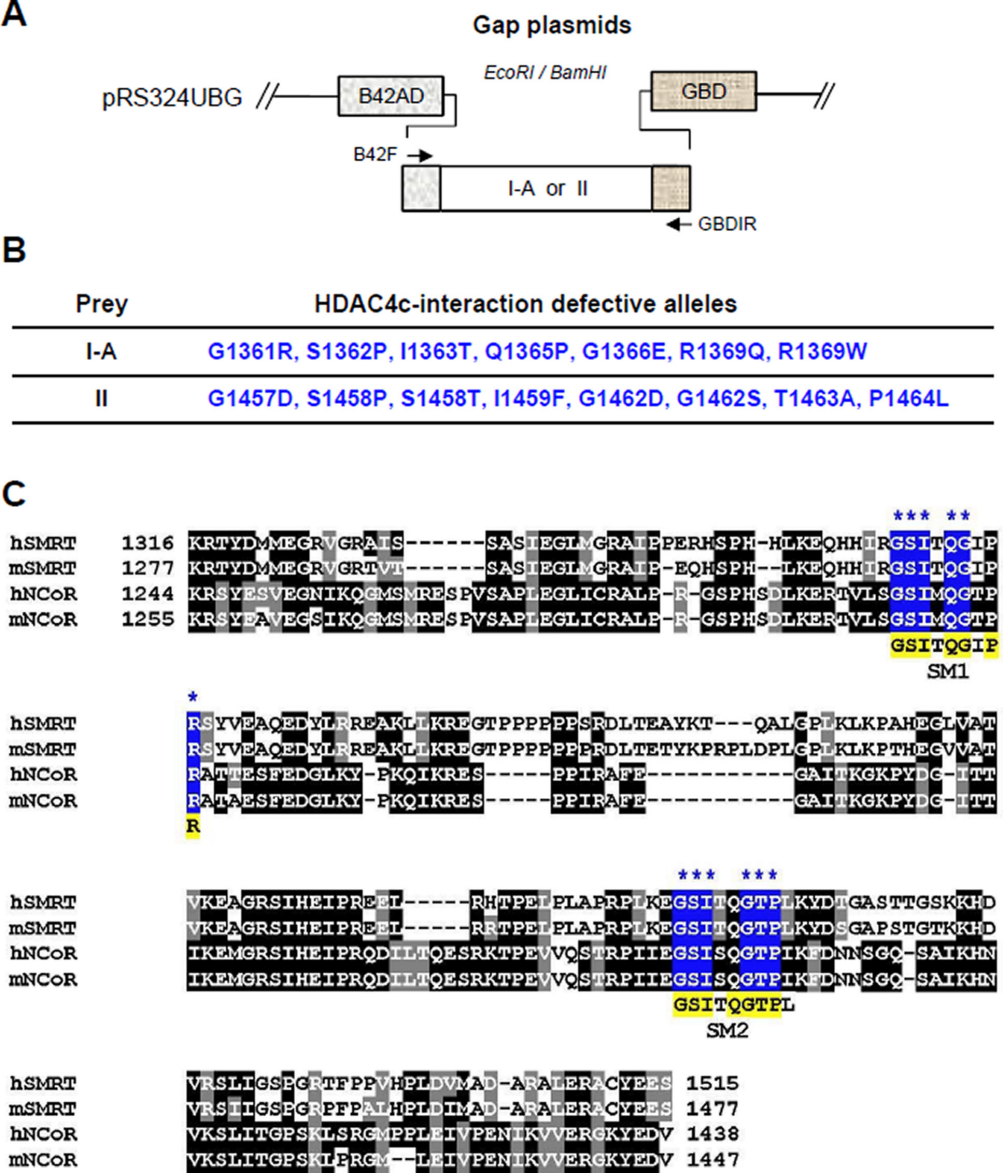
OPTHiS screening identifies two SRD3c conserved motifs required for HDAC4c binding. (**A**) Schematic diagram of the prey constructs used for OPTHiS screening. Randomly mutagenized I-A or II fragments were inserted between the B42AD and the GBD regions of gap plasmid to construct the prey mutant library for OPTHiS screening. (**B**) Positions and amino acid changes of isolated I-A or II mutant alleles defective for HDAC4c binding. (**C**) Multiple sequence alignments of RD3c regions of SMRT and NCoR proteins from *H. sapiens* (h), *M. musculus* (m). Sequence alignment was performed and presented using the ClustalW and Boxshade programs, respectively. Positions and amino acids of the isolated mutations are marked with stars and shaded in blue. Conserved amino acids within SM1 and SM2 motifs are shaded in yellow.

The plasmids pRS324UBG-HDAC4c/-5c, KGC-MC-HDAC4c/-5c, pcDNA3-HA-HDAC4c/-5c, and KGN-MC-SRD3c were described previously (20). To construct expression vectors for HDAC4c derivatives, the amplified human HDAC4c region (E613–L1084) was inserted into the BamHI/NotI sites of the pRS325LexA vector [pRS325LexA-HDAC4c to be used in one-plus two-hybrid system (OPTHiS)] and ClaI/NotI sites of the pEBG vector (pEBG-HDAC4c for *in vivo* glutathione *S*-transferase (GST) pull-down). To prepare the prey plasmid pRS324UBG series, the amplified SRD3c or deletion constructs (I, I-A, I-B, I-C, or II) was inserted into the EcoRI/BglII sites of pRS324UBG (Figure 1A). To construct expression vectors for LexA- or GST-fused SRD3c (K1316-S1515 of human SMRT), SMRT motif-1 (SM1F, E1346-L1385), or SMRT motif-2 (SM2F, H1423-R1482), each of the amplified DNA fragments was inserted into the EcoRI/XhoI sites of pEG202 or pGEX4T-1 (Amersham Biosciences, Amersham, UK), respectively. To make pCMX-Gal4N-SRD3c, the amplified SRD3c region was inserted into the EcoRI/SalI sites of the pCMX-Gal4N vector. All SM1 or SM2 mutants isolated by OPTHiS were introduced into the SM1F or -SM2F context in pEG202 (for yeast two-hybrid assay), pGEX4T-1 (for GST pull-down assay), or KGN-MC (for BiFC assay), respectively, by PCR amplification or enzyme digestion from the pRS324UBG version. To introduce point mutation(s) into pGEX4T-1-SRD3c (mt1, mt2, and dmt), pGEX4T-1-SM1F (R+9L), or pGEX4T-1-SM2F (L+9R), site-directed mutagenesis was performed using a Quickchange II site-directed mutagenesis kit according to the manufacturer's instruction (Agilent Technologies, Santa Clara, CA, USA). All mutants were subcloned into the pEG202 or KGN-MC vectors at the EcoRI/XhoI or BamHI/XhoI enzyme sites, respectively. For structural analysis, the gene for the human HDAC4c (R651–T1055) H976Y mutant was amplified by PCR using the human HDAC4c H976Y clone (E613–L1084) as a template and primers designed for ligation-independent cloning (LIC) (24). The amplified PCR product was annealed into the LIC expression vector pB3, a derivative of pET21a (Novagen, Madison, WI, USA). All constructs were confirmed by DNA sequencing.

### Mutagenic PCR and OPTHiS screening

Random mutagenesis and OPTHiS screening of I-A or II fragments were conducted as described previously (25). OPTHiS is the modified version of the conventional yeast two-hybrid system for efficient selection of missense mutant alleles that specifically disrupt a known protein-protein interaction. As a first step in isolating the full-length missense alleles of the I-A or II fragments, mutagenic PCR fragments containing these regions were amplified using pRS324UBG-I-A or -II as PCR templates, respectively. Next, the mutagenic PCR products (800 ng) were co-transformed with linearized gap plasmids (200 ng) into the yeast strain YOK400 (*MATa, leu2, trp3, ura3*,*lexA_op_-LEU2, UAS_GAL_-HIS3*) carrying the pSH18-34 reporter and the bait plasmid pRS325LexA-HDAC4c. During yeast transformation, a gap repair process occurred in the linearized gap plasmids using I-A or II fragments as templates, resulting in one-step construction of a mutant cell library for these regions. The transformants were grown at 30°C for 3 days on synthetic glucose medium lacking histidine for positive selection of intact prey fusion (B42- I-A/II-GBD) using the endogenous *UAS_GAL_-HIS3* reporter gene. In the second step of screening of the non-interactor, 3500–4000 transformants were picked from X-gal plates to select for interaction-defective mutants (white colonies). Candidates colonies were subjected to a verification step as described previously (25).

### Yeast two-hybrid assay and *in vitro* GST pull-down assay

The yeast strain EGY48 containing pSH18-34 (8X*lexA_op_-LacZ* reporter plasmid) was co-transformed with expression plasmids for LexA-fused bait and B42-GBD-fused prey by the lithium acetate method. Liquid assays for β-galactosidase activity were carried out for three transformants as described previously (25). HA-tagged wild-type HDAC4c/-5c was labeled with ^35^S-methionine using a TNT *in vitro* translation kit (Promega, Madison, WI, USA). The radiolabeled proteins were mixed with equivalent amounts of GST or GST-fused proteins bound to glutathione-agarose beads (Sigma-Aldrich, St. Louis, MO, USA) pre-equilibrated with GST binding buffer [25 mM HEPES–KOH (pH 7.5), 10% glycerol, 200 mM KCl, 1 mM EDTA, 0.01% Nonidet P-40, 1 mM DTT, 1× protease inhibitor cocktail] in a final volume of 300 μl. The beads were washed three times with the same buffer, and bound proteins were analyzed by 8% SDS-PAGE followed by autoradiography. Details of the expression and purification of GST proteins have been described previously (20).

### Cell culture and transient transfection assay

HEK293 cells were maintained in DMEM (Welgene, Daegu, Korea) supplemented with 10% fetal bovine serum (Welgene) and antibiotics-antimyces (Gibco, Grand Island, NY, USA). Cells were seeded into 24-well plates with 5–8 × 10^4^ cells/well on the day prior to transfection. Transient transfections were performed using the TurboFect (Fermentas, Waltham, MA, USA) systems, as described in the manufacturer's instructions. After 24 h of transfection, whole-cell lysates were prepared in RIPA buffer [50 mM Tris–HCl (pH 8.0), 5 mM EDTA, 150 mM NaCl, 1% NP-40, 1 mM PMSF] and used for the BiFC assay.

### BiFC assay and confocal laser-scanning microscopy

To examine the protein/protein interactions of HDAC4c/-5c with SRD3c, SM1F, or SM2F derivatives in mammalian cells, bimolecular fluorescence complementation (BiFC) assays were carried out using a Fluo-Chase kit (Amalgaam). The BiFC assay is a novel assay method for evaluating protein-protein interactions in living cells and is independent of the transcription system. First, we constructed expression plasmids for the N-terminal portion of the Kusabira Green protein (KGN) fused with SRC3c/SM1F/SM2F derivatives as well as the C-terminal portion of Kusabira Green (KGC) fused with HDAC4c/-5c. If the two proteins interact, a green fluorescence signal is generated from the reconstituted Kusabira Green protein. After 48 h of transfection of these expression constructs in HEK293 cells, whole-cell lysates were prepared and fluorescent signals (excitation wavelength: 494 nm, emission wavelength: 538 nm) from the samples were measured using a fluorescence spectrometer (Spectra max GEMINIXPS, Molecular Devices, San Jose, CA, USA). For confocal laser scanning microscopy, HEK293 cells were grown on 8-well slide plates (SPL Life science, Gyeonggi-do, Korea) and co-transfected with expression vectors for KGN-fused and KGC-fused constructs. After 48 h of transfection, the cells were incubated in 4% paraformaldehyde for 10 min at room temperature for fixation. The cells were then washed with 1× PBS, mounted onto micro cover-slides, and observed for green fluorescence using a laser-scanning confocal microscope (Leica TCS SPE, Wetzlar, Germany).

### *In vivo* GST pull-down assay

GST-tagged HDAC4c was coexpressed with Gal4N-fused SRD3c wild-type or mutants in HEK293 cells. After 48 h, the whole cell lysates (500 μg) were prepared in RIPA buffer [50 mM Tris-HCl (pH 8.0), 5 mM EDTA, 150 mM NaCl, 1% NP-40, 1 mM PMSF] and mixed with 30 μl (50% slurry) of glutathione-agarose beads (Sigma-Aldrich). After overnight incubation at 4°C, the beads were washed three times with the same buffer. The bound proteins were eluted from the precipitates using 2× SDS sample buffer and resolved by 10% SDS-PAGE. The presence of Gal4N-SRD3c proteins and GST- HDAC4c was analyzed by immunoblotting using anti-GBD (sc-510; Santa Cruz Biotechnology, Dallas, TX, USA) and anti-GST (sc-459; Santa Cruz Biotechnology) antibodies, respectively.

### Expression and purification of human HDAC4c for structural studies

The recombinant human HDAC4c (R651-T1055) of H976Y mutant was expressed in an *Escherichia coli* BL21 Star (DE3) strain (Novagen). Cells were cultured in LB medium containing 100 μg/mL ampicillin at 37°C and then treated with 0.1 mM Isopropyl β-D-1-thiogalactopyranoside for an additional 24 h at 18°C. The harvested cells were re-suspended and disrupted by ultrasonication in ice-cold buffer solution [20 mM Tris-HCl (pH 7.5), 500 mM NaCl, 30% glycerol, 10 mM β-mercaptoethanol (βME), 0.5% NP-40]. The supernatant was loaded onto a 5-mL HisTrap chelating column (GE Healthcare, Little Chalfont, UK) and the column was extensively washed with buffer A [20 mM Tris–HCl (pH 7.5), 500 mM NaCl, 10% glycerol, 10 mM βME, 0.1% NP-40]. The bound protein was eluted with a linear gradient from 0 to 500 mM imidazole in buffer A. The resulting protein was digested by a recombinant Tobacco etch virus protease to remove the 6xHis-tag, and then dialyzed against buffer B [20 mM Tris–HCl (pH 7.5), 200 mM NaCl, 10% glycerol, 10 mM βME, 0.1% NP-40]. The protein was further isolated by using a HiTrap Q anion exchange column (GE Healthcare) operated over a linear gradient from 0 to 1 M NaCl in buffer B.

The human SMRT peptides were chemically synthesized (Anygen, Gwangju, Korea) and dissolved in a buffer [20 mM Tris–HCl (pH 7.5), 200 mM NaCl]. To generate complexes of HDAC4c H976Y and peptides, the protein was mixed with each peptide at a molar ratio of 1 to 2 in a solution [20 mM Tris-HCl (pH 7.5), 200 mM NaCl, 10 mM βME, 0.1% *n*-octyl-β-d-glucoside] and incubated at 4°C for 6 h.

### Crystallization, data collection, and structure determination

HDAC4c H976Y-peptide complex was concentrated to 20 mg/ml and used for crystallization attempts at 22°C by the sitting-drop vapor-diffusion method. Initial crystallization hits were optimized by a grid search using 24-well Linbro plates and the hanging-drop vapor-diffusion method at 22°C, where 2 μl protein and 1.4 μl reservoir solution were mixed and equilibrated with 0.2 ml precipitant containing 12.5% polyethylene glycol 3350, 0.1 M Hepes-Na (pH 7.5), and 8% isopropanol. Crystals were briefly immersed in precipitant solution containing an additional 25% of 2-methyl-2,4-pentanediol as the cryo-protectant and immediately placed in a 100 K liquid nitrogen-gas stream. Diffraction data were collected at the 5C beam line of the Pohang Accelerator Laboratory (PAL, Korea) with a per-frame oscillation of 1° and per-frame exposure of 10 s. A total of 180 images of each complexed crystal were collected on the ADSC-Q270 CCD detector. Indexing, integration, and scaling of the reflections were conducted using the *HKL*2000 suite (26).

The crystal structure was solved by the molecular replacement program Phaser-1.3 (27) using the human HDAC4c structure (PDB ID 2vqw) as a search model. Further model building was performed manually using WinCoot (28), and subsequent refinement was performed with PHENIX (29). The data and refinement statistics are summarized in Table 1. The quality of the model was analyzed by using MolProbity (30).

**Table 1.** Data collection and structure refinement statistics

Data collection	SP1-HDAC4c	SP2-HDAC4c
Space group	*P*3_1_21	*P*3_1_21
Unit cell dimensions		
*a, b, c* (Å), α, β, γ (°)	131.42, 131.42, 67.77, 90, 90, 120	132.0, 132.0, 67.79, 90, 90, 120
Wavelength (Å)	0.9793	1.0000
Resolution (Å)	30–1.85 (1.88–1.85)^a^	50–2.70 (2.75–2.70)^a^
*R*_sym_	13.8 (45.0)	13.9 (45.4)
*I*/σ(*I*)	12.0 (2.6)	7.0 (1.9)
Completeness (%)	98.5 (97.8)	95.6 (91.8)
Redundancy	3.2 (3.2)	2.9 (2.5)
**Refinement**		
No. of reflections	57039	18174
*R*_work_/*R*_free_	15.6 (22.6)/17.4 (24.3)	21.6 (29.3)/23.5 (29.4)
No. atoms protein/water/Zn/K	3038/279/2/2	2991/118/2/2
R.m.s. deviations bond lengths (Å)/angles (°)	0.004/0.71	0.002/0.57
Average *B*-values (Å^2^) protein/water	31.5/42.2	36.3/ 29.8
Ramachandran plot (%)		
favored/allowed/outliers	97.0/2.8/0.2	97.4/2.3/0.3

^a^The numbers in parentheses are the statistics from the highest resolution shell.

### Isothermal titration calorimetry (ITC)

ITC measurements were carried out with the purified human HDAC4c (R651-T1055) of H976Y mutant and synthesized peptides dissolved in the same buffer [20 mM Tris–HCl (pH 7.5), 200 mM NaCl, 0.1% NP-40, 0.5 mM TCEP]. ITC measurements were performed at 20°C using a Nano ITC calorimeter (TA Instruments, New Castle, DE, USA). NanoAnalyze software (TA Instruments) was used to study the binding isotherms. Baseline controls were acquired in buffer and the peptides were measured in the same buffer. Titrations were performed as a set of 25 injections of each peptide (2.25 mM) into HDAC4c (0.113 mM). Fittings were performed using the independent binding sites model.

## RESULTS

### Two conserved motifs within SRD3c are required for HDAC4c/-5c binding

A previous bioinformatics approach revealed several GSI motifs within the RD3 regions of SMRT/NCoR as the molecular determinants of their interactions with HDAC4c/-7c (19,31). However, these studies did not determine whether these motifs are essential for the interactions or evaluate which motif is important for the interactions. Thus, we tested the binding strength of the N-terminal region (SRD3n, R1128–E1289) and C-terminal region (SRD3c, R1317–S1515) of SRD3 to HDAC4c/-5c in yeast two-hybrid, GST pull-down, and BiFC assays. We observed a marginal interaction between SRD3n and HDACs (Supplementary Figure S1), which is consistent with previous results (18). We also examined the contribution of putative GSI motifs of SRD3T (R1128–K1592) to its binding to HDACs, indicating that GSI motifs within the SRD3c region function as major binding elements (Supplementary Figure S1).

Next, we targeted the SRD3c region (R1317–S1515) and mapped the region(s) for HDAC4c binding using deletion constructs (I, I-A, I-B, I-C, and II) in yeast two-hybrid assays (Supplementary Figure S2). This experiment revealed that two regions of SRD3c (I-A: K1316–G1390, II: P1396–S1515) independently interacted with HDAC4c/-5c, although the binding strength of region II was relatively weaker than that of region I-A. These regions were evaluated to identify the amino acids essential for the HDAC4c interaction, resulting in the isolation of 7 and 8 I-A and II mutant alleles, respectively (Figure 1A and B). Interestingly, all mutant alleles were mapped to the conserved GSI motif sequence (GSIxQGxPx) as the mutational hot spot in both fragments (Figure 1C), confirming previous observations that GSI motifs are critical determinants for SRD3c binding to class IIa HDACs (19,31). We named these two motifs as SM1 (^1361^GSITQGIPR^1369^) and SM2 (^1457^GSITQGTPL^1465^) to indicate SMRT motif-1 and SMRT motif-2 and denoted the first glycine of the SM1 (GSITQGIPR) or SM2 (GSITQGTPL) as +1 for convenience. The importance of the isolated SM1 and SM2 mutants in HDAC4c/-5c/-7c binding was confirmed by yeast two-hybrid, GST pull-down, and BiFC interaction assays (Supplementary Figures S3–S5).

### SM1 is more important than SM2 for the interaction between SRD3c and HDAC4c/-5c

**Figure 2.**
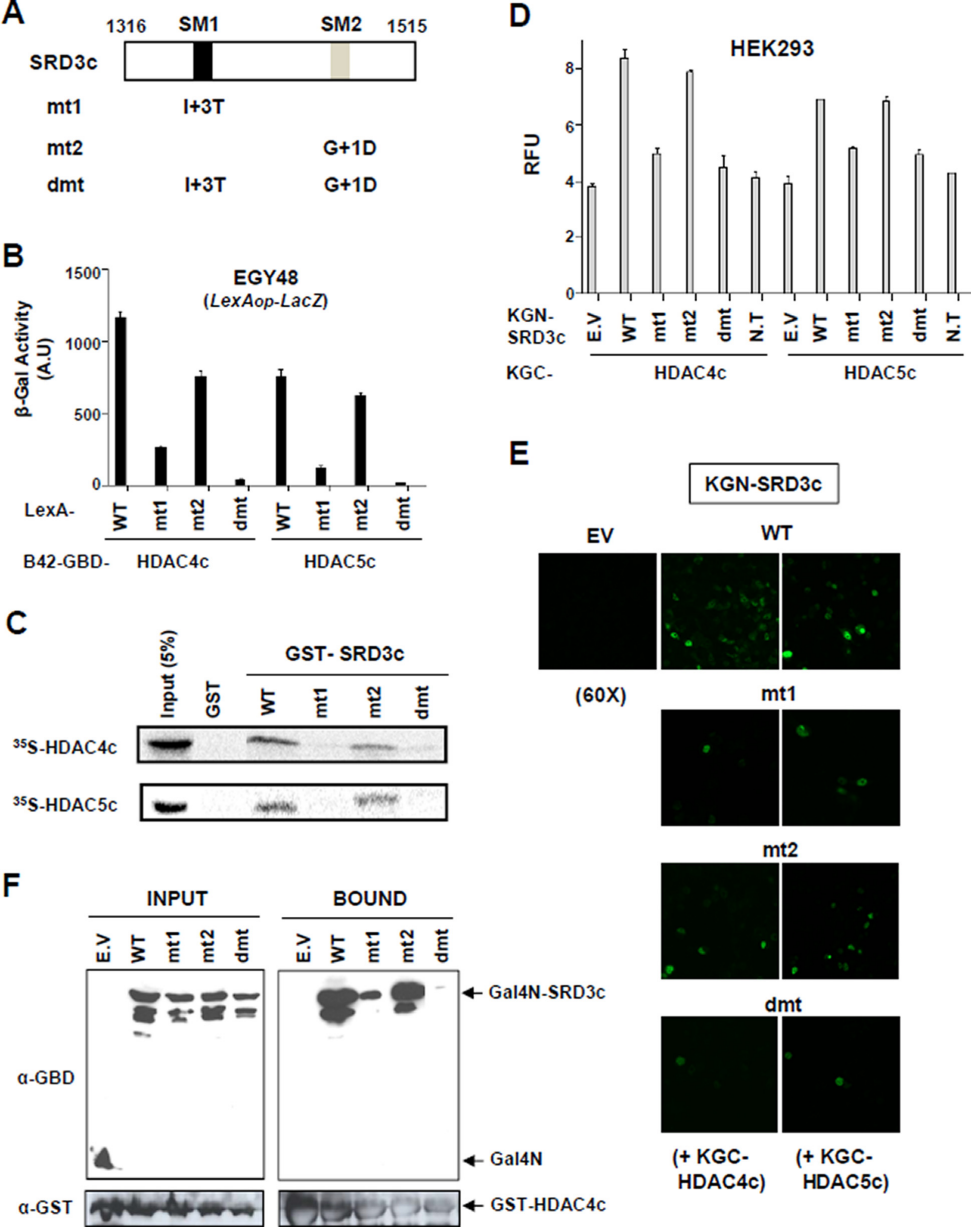
SM1 is more important than SM2 for the interaction between SRD3c and HDAC4c/-5c. (**A**) Schematic diagram of SRD3c mutants. The first glycine residue of the SM1 (GSITQGIPR) or SM2 (GSITQGTPL) is denoted as +1. Single amino acid substitution is introduced into SM1 (I+3T) or SM2 (G+1D) motif of SRD3c and denoted as mt1 or mt2, respectively. The dmt indicates double-mutant containing both mt1 and mt2 mutations. (**B**) Yeast two-hybrid assay. Yeast strain EGY48 bearing *lexA_op_-LacZ* reporter was co-transformed with expression plasmids for LexA-fused SRD3c derivatives (wild-type, mt1, mt2, or dmt) and B42AD-GBD fusions of HDAC4c/-5c. Transformants were grown in synthetic minimal glucose media, and liquid β-galactosidase assays were performed. WT: wild-type. (**C**) *In vitro* GST pull-down assay. GST or GST-SRD3c derivatives were tested for interactions with *in vitro* synthesized HDAC4c/-5c proteins. Input represents 5% of the *in vitro* translated HDAC4c/-5c used in the pull-down assays. (**D**) Quantitative BiFC assay in HEK293 cells. Fluorescence signals generated via the interactions between indicated KGN-SRD3c proteins and KGC-HDAC4c/-5c were spectrophotometrically measured as described in Materials and methods. (**E**) Confocal laser-scanning image for the BiFC interaction assay between indicated KGN-SRD3c proteins and KGC-HDAC4c/-5c. *Magnification*: 60 X. (**F**) *In vivo* GST pull-down assay. HEK293 cells were transiently transfected with expression vectors for GST-HDAC4c and indicated Gal4N-SRD3c derivatives. Whole-cell extracts were prepared and incubated with glutathione-Sepharose beads. The bound proteins were analyzed via immunoblotting using anti-GBD (for Gal4N) and anti-GST antibodies. E.V: empty vector, N.T: no transfection.

We constructed the SM1F and SM2F fragments, which correspond to the 40-amino acid fragment containing SM1 or SM2 in the middle, respectively (Supplementary Figure S6A). The SM1F and SM2F independently interacted with HDADC4c/-5c/-7c in all assay systems tested (Supplementary Figures S5 and S6) with better binding activity of SM1F than SM2F to all HDACs. Next, we introduced single- (mt1, mt2) or double-mutations (dmt) into the SM1 or/and SM2 motifs of SRD3c (Figure 2A) and examined the effects of the mutations on HDAC4c/-5c binding using various assay systems (Figure 2B–F). Notably, the mt1 mutation showed a more deleterious effect on HDAC binding than mt2. Taken together, our data consistently indicate that SM1 is more important than SM2 for SRD3c binding to HDAC4c/-5c/-7c (Figure 2 and Supplementary Figure S5A).

We also generated full-length SMRT mutants with mt1, mt2, or dmt mutations via *in vitro* translation and investigated their HDAC-binding activities in a GST pull-down assay. However, we obtained no positive results because of the low expression level of full-length SMRT protein and its non-specific binding activities (data not shown).

### Structures of HDAC4c in complex with two SMRT peptides

**Figure 3.**
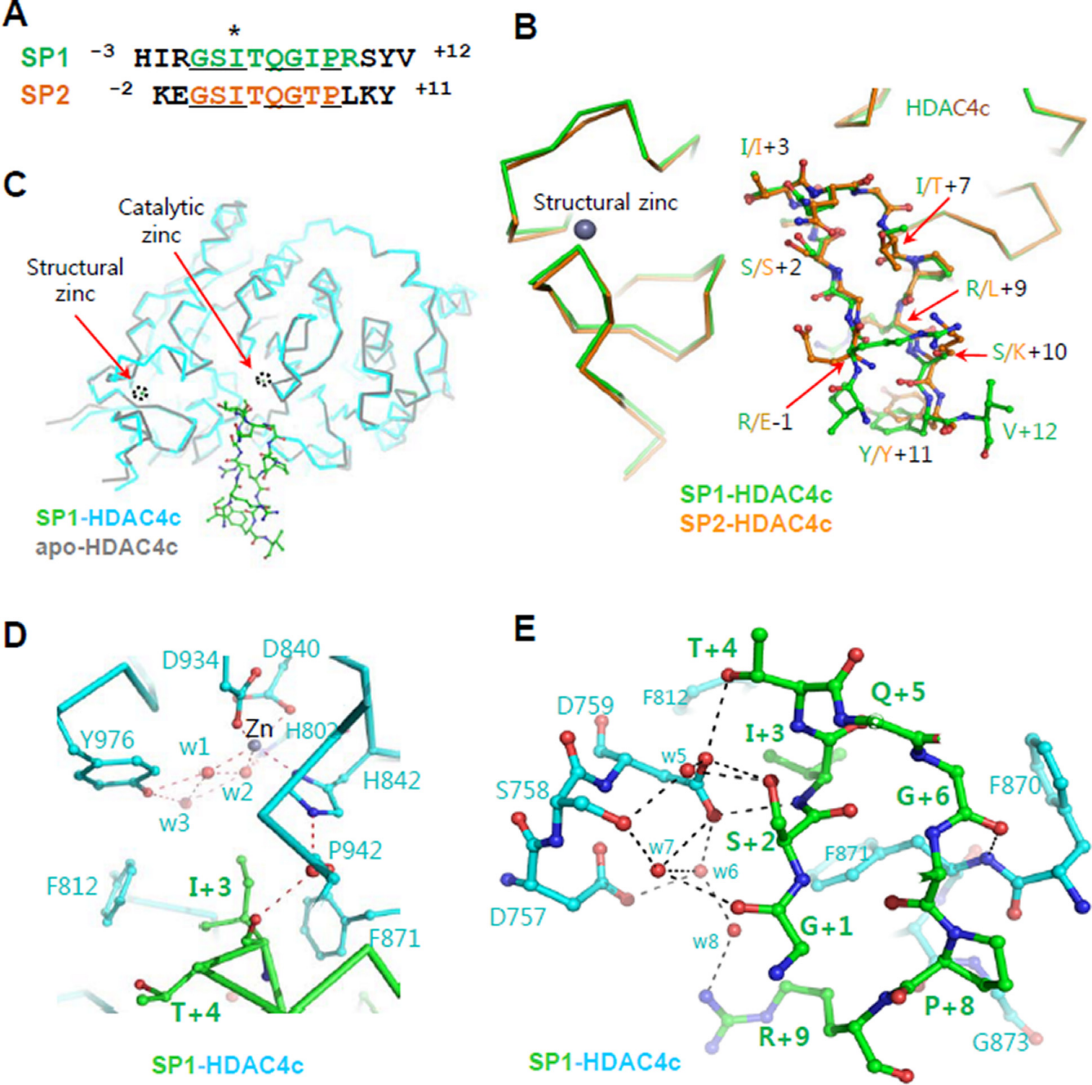
HDAC4c structures in complex with SMRT peptides. (**A**) Sequence alignment of SP1 and SP2 peptides derived from two SMRT consensus motifs. The I+3 residues (I1363 and I1459) sandwiched between the two conserved phenylalanine residues (F812 and F871) of HDAC4c are indicated by an asterisk. The strictly conserved residues are underlined. (**B**) Superposed structures of HDAC4c in complex with two peptides. HDAC4c and bound peptides are shown in different colors. Peptide side-chains are displayed with the ball-and-stick models. The structural zinc ion is drawn as a black sphere. (**C**) Superposed structures of closed conformation (gray) and the peptide complex (cyan-green). The catalytic and structural zinc ions are drawn as spheres and indicated with black-dotted circles. (**D**) A close-up view of the catalytic zinc ion-binding site in the peptide-complex structure. The zinc ion and water molecules are drawn as gray and red spheres, respectively. The residues of HDAC4c (cyan) and bound SP1 peptide (green) are displayed with the ball-and-stick models. The interactions among atoms are displayed by red-dotted lines. (**E**) Interaction of SP1 peptide with HDAC4c at the cleft to the catalytic site. Water molecules are displayed with red spheres and interacting residues are presented with the ball-and-stick models. Polar interactions among atoms are indicated with black-dotted lines.

To determine the mechanistic basis of how class IIa HDACs are targeted and regulated by the SMRT corepressor, we performed a structural study of the HDAC4c-SRD3c complex (Figure 3). For the SRD3c protein, we designed two peptides (SP1: ^−3^HIRGSITQGIPRSYV^+12^, SP2: ^−2^KEGSITQGTPLKY^+11^), which include the GSI motif in the sequences. For kinetic analysis of peptide binding to HDAC4c H976Y (R651-T1055), we performed ITC experiments and obtained a dissociation constant of 2.23 μM for SP1 and 18.8 μM for SP2 (Table 2 and Supplementary Figure S7). This result confirmed our previous finding that SM1 is more important than SM2 for SRD3c binding to class IIa HDACs. Consistent with this, these peptides inhibited the *in vitro* deacetylase activity of HDAC4c H976Y, with a better inhibitory effect of SP1 than that of SP2 (Supplementary Figure S8A). However, these peptides did not inhibit HDAC8 activity under the same conditions, indicating the specific binding of GSI motifs to class IIa enzymes (Supplementary Figure S8B).

**Table 2.** Thermodynamic parameters for the binding of SP1 and SP2 to HDAC4c

Thermodynamic parameters	SP1-HDAC4c	SP2-HDAC4c
*K*_d_ (μM)	2.23	18.8
Δ*H*° (kJ/mol)	−47.01	−29.35
Δ*S*° (J/mol·K)	−52.16	−9.63
Stoichiometry	0.820	0.977

The complex structures were determined by a molecular replacement method using the apo-form HDAC4c structure (PDB ID 2vqw) (21) as an initial search model and each peptide-complex structure was refined at the defined resolutions, respectively (Table 1). Similar to the reported HDAC4c structures (21,23), the HDAC4c-peptide complex structures lack an interpretable electron density for residues (L728–S744) around the structural zinc ion-binding site. Overall, the refined HDAC4c-peptide structures superpose well on the apo-form structure, indicating that peptides binding did not rearrange the catalytic and structural zinc ion-binding sites (Figure 3C and Supplementary Figure S9). Two loops of S744–W762 and H803–G811 in the structural zinc-binding subdomain (F652–N763), along with residues in the R864–P877 loop of the C-terminal catalytic subdomain (E764–T1055), form a cleft wall leading to the catalytic site. The catalytic zinc ion is coordinated with six atoms: four protein atoms from the side-chains of residues D840, H842, and D934, and two from the two water molecules (w1 and w2) which further interact with the hydroxyl group of Y976 through another water molecule w3 (Figure 3D) (Supplementary Table S1). The bound peptides commonly form a β-hairpin structure of two β-strands in the HDAC4c cleft leading to the catalytic zinc ion (Figure 3B). The hydrophobic I+3 residues (I1363 or I1459) are at the β-turn near the catalytic zinc ion and intervened with F812 and F871 at the end of the cleft and entrance to the catalytic pocket of the HDAC4c (Figure 3B–D).

There are several water-mediated or direct polar interactions between the bound peptides and HDAC4c (Supplementary Table S2). At one side of the cleft, a water molecule (w4) mediates the interaction between the carbonyl oxygen of I+3 in both peptides with the side-chain of H842 of the catalytic subdomain (Figure 3D). At the two loops of S744–W762 and H803–G811 of the structural zinc ion-binding site, D759 hydrogen-bonds with the hydroxyl group of T+4 and peptidyl nitrogen atom between S+2 and I+3 in two peptides (Figure 3E). Other water molecules (w5–w8) also mediate the interaction between the bound peptide and structural zinc-binding subdomain (Supplementary Table S2). Notably, D757 interacts indirectly with R+9 of the bound SP1 via a water molecule (w8, Figure 3E), which is missing in the SP2-bound structure with L+9 at the corresponding position (Figure 3B).

### Analysis of structure-based SM1 mutants

**Figure 4.**
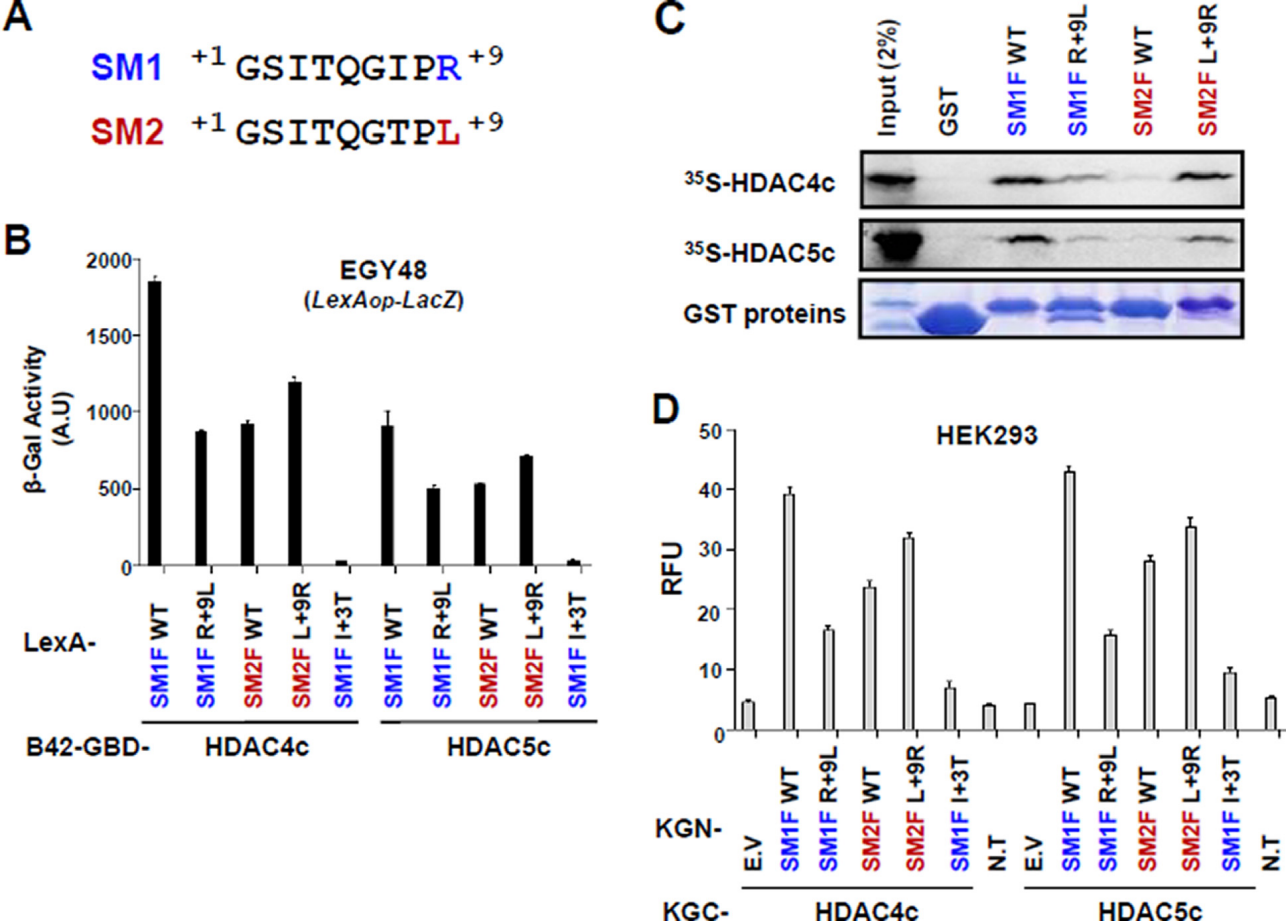
SM1 R+9 is important for optimal interaction of SM1 fragment with HDAC4c/-5c. (**A**) Comparison of SM1 and SM2 sequences. (**B**) Yeast two-hybrid interactions of indicated LexA-fused SM1F/-2F proteins with B42-GBD-HDAC4c/-5c. (**C**) *In vitro* GST pull-down assay for the interactions of GST or GST-SM1F/-2F derivatives with *in vitro* synthesized HDAC4c/-5c proteins. GST proteins were resolved by 12% SDS–PAGE and visualized via Coomassie Brilliant blue staining. (**D**) Quantitative BiFC assay for the interaction of KGN-SM1F/-2F mutants with KGC-HAC4c/-5c proteins in HEK293 cells. WT: wild-type, E.V: empty vector, N.T: no transfection.

Our results indicate that SM1 has a stronger affinity in SRD3c binding to HDACs compared to SM2 (Figure 2, Table 2, and Supplementary Figure S6). Sequence comparison of SM1 and SM2 suggested that the I+7 or R+9 residues of SM1 are responsible for this difference. According to the determined structures, the SP1 R+9 residue interacts with the structural zinc-binding region (although this interaction is water-mediated), and SP1 I+7 and SP2 T+7 are exposed to solvent without interacting with the protein (Figure 3B and E), suggesting the greater importance of the SM1 R+9 residue than the I+7 residue. To test this prediction, we made swap mutants between SM1 R+9 and SM2 L+9 residues and evaluated their interactions with HDAC4c/-5c/-7c (Figure 4 and Supplementary Figure S5D). Interestingly, HDAC-binding activity was significantly decreased in the SM1 R+9L mutant (SM2 mimic), whereas the SM2 L+9R mutant (SM1 mimic) exhibited increased binding activity.

**Figure 5.**
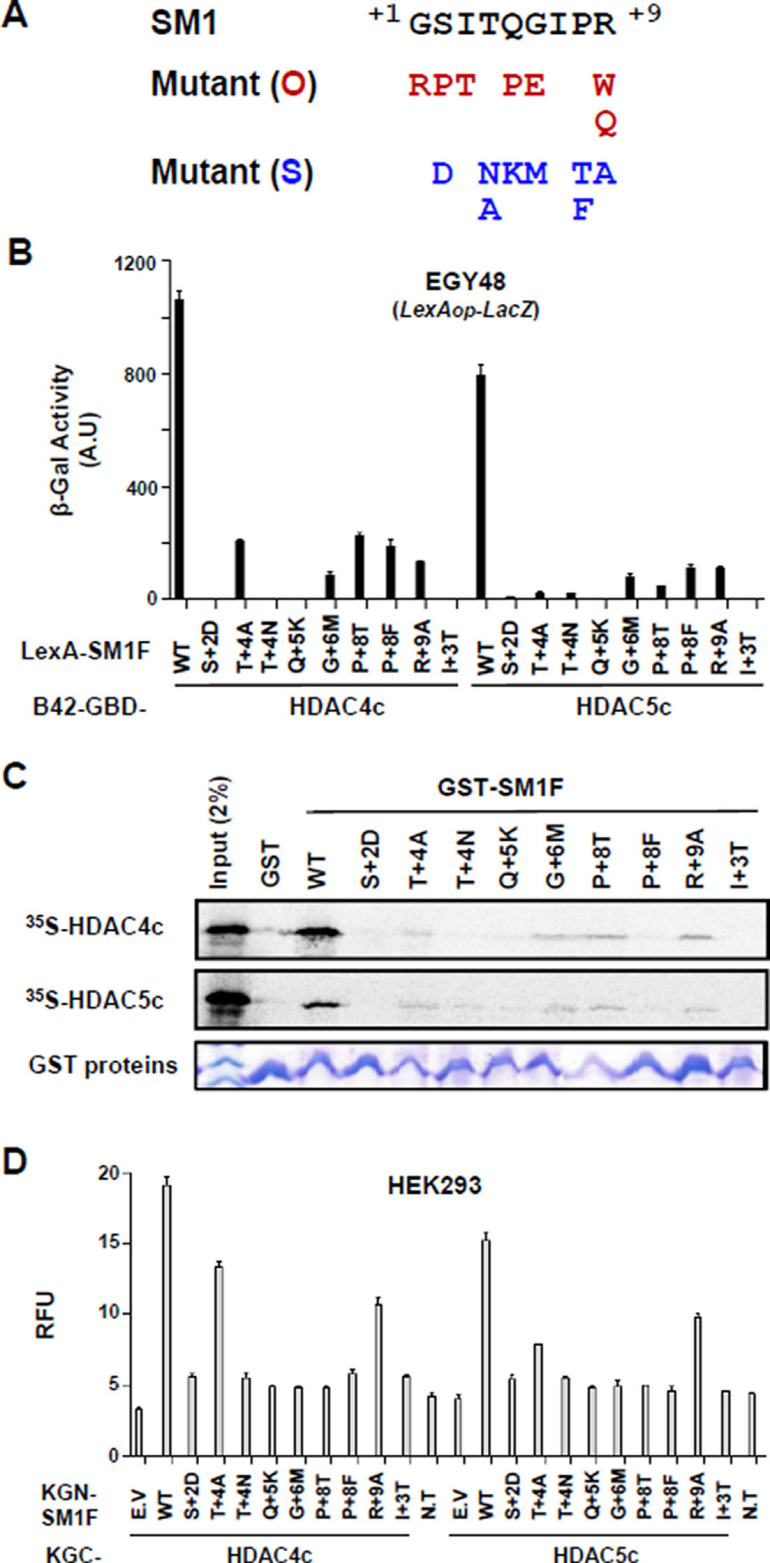
Interactions of HDAC4c/-5c with SM1 mutants generated based on structural information. (**A**) SM1 mutant alleles obtained via OPTHiS screening (O) or site-directed mutagenesis (S) are shown in red and blue colors, respectively. SM1(S) mutant alleles were designed based on the HDAC4c-SP1 structure. (**B**) Yeast two-hybrid assay. LexA-fused SM1F containing indicated SM1(S) mutation was tested for B42-GBD-HDAC4c/-5c in EGY48 strain. (**C**) *In vitro* GST pull-down assay. *In vitro* synthesized HDAC4c/-5c were examined for their binding to GST or GST-fused SM1F derivatives. (**D**) Quantitative BiFC assay for the interaction between indicated KGN-SM1F mutants and KGC-HAC4c/-5c proteins in HEK293 cells. WT: wild-type, E.V: empty vector, N.T: no transfection.

Next, we analyzed the mutational effects of SM1 mutants by OPTHiS screening (Figure 5A). First, the isolation of I+3T mutant showed that the hydrophobic character at this spatial position is necessary for the interaction with two conserved phenylalanine residues of both HDACs (Supplementary Figure S3). In the peptide-complex structures, G+1, S+2 and T+4 residues on both peptides interact, directly or through water molecules, with residues on the cleft wall of the structural zinc-binding subdomain (Figure 3E). Mutations in this peptide region (G+1R, S+2D and T+4N/T+4A) altered the binding affinity of the SM1F fragment to HDAC4c. Introduction of large residues (G+1R and S+2D) may interrupt, due to steric repulsion, the observed interaction between the peptide side-chain atoms and the structural zinc-binding loops of HDAC4c (Figure 3B and E). The abrogated interactions accompanying the mutation at the T+4 site (T+4N/T+4A) of SM1 or at HDAC4c D759 (D759Y) indicate that the observed hydrogen bond between D759 and T+4 is necessary for their interaction (Figure 3E). Similarly, a mutational effect at SM1 R+9 (R+9W/R+9Q/R+9A) was found to be important for optimal binding to the structural zinc-binding subdomain of HDAC4c/-5c/-7c, as suggested previously.

Further analysis confirmed that sequence conservation in SM1 is required for the specific binding to both HDACs *in vivo* and *in vitro* (Figure 5). Mutations introduced at the G+6 and P+8 positions (G+6M and P+8T/P+8F) of SM1 affected the binding affinity of the SM1F to HDACs, indicating that the formation of the β-hairpin structure with a rigid second β-sheet, shown in peptide structures, is essential for SRD3c binding to HDACs. The Q+5 side-chain is completely exposed to solvent in both peptide-complex structures (Figure 3B and E). Why the Q+5K mutation abolished SM1F binding to the protein based on the current structure remains unclear. Taken together, these data confirm that the spatial positioning of peptides at the cleft wall of the structural zinc-binding subdomain in HDAC4c/-5c and formation of a β-hairpin structure with an isoleucine residue at the β-turn are critical for SRD3c binding to both HDACs.

## DISCUSSION

### β-hairpin structure and R+9 residue of SM1 are major determinants of SRD3 binding to class IIa HDACs

Although a previous bioinformatics approach identified several GSI-containing motifs within SRD3 as the molecular determinants of the interactions with HDAC4c/-7c, these studies did not determine which motif(s) is (are) essential for SRD3-HDAC interactions (19,31). In this study, we evaluated the molecular determinants responsible for the SRD3 interaction with HDAC4c by domain mapping and OPTHiS screening, and found that two conserved GSI motifs of SRD3c (SM1 and SM2) are critical for SRD3c binding to HDAC4c/-5c (Figure 1). In the structures, the GSI regions of the first β-strand of the β-hairpin structure in SP1 and SP2 directly interact with the structural zinc-binding subdomain, while the residues of the second β-strand interact with the catalytic subdomain (Figure 3). The two β-strands in the bound peptides can also be discriminated by atoms involved in hydrophilic interactions. The first β-strand of peptides uses its side-chain atoms, while the second β-strand uses its main-chain atoms, except for the interaction at the R+9 residue in SM1 (Figure 3D and E). Interestingly, our mutational analysis indicated that SM1 has a more important role than SM2 in SRD3c binding to HDAC4c/-5c/-7c (Figure 2 and Supplementary Figure S5A). The two motifs are very close to each other, with a major difference in the structurally relevant positions R+9 in SM1 and L+9 in SM2 (Figure 3A and B). In this regard, an SM2-mimic SM1 derivative (R+9L) showed significantly decreased binding activity to HDACs (Figure 4 and Supplementary Figure S6D). The reverse SM2 mutant, an SM1-mimic L+9R mutant, showed increased binding activity to HDACs, which was comparable to that of wild-type SM1. These results suggest that formation of a β-hairpin structure along with an R+9 residue at the end of the second β-strand in SP1 is a major contributor to the SRD3c interaction with HDAC4c/-5c/-7c. ITC assays also consistently revealed higher binding affinity of SP1 to HDAC4c H976Y than that of SP2 by ∼9-fold (Table 2).

### Closed conformation of HDAC4 is required for SRD3 binding

Analysis of the reported HDAC4c structures revealed remarkable conformational change around the structural zinc ion-binding site. In the apo-form structure, two loops, S744–W762 and H803–G811, construct a cleft wall at one side, leading to the catalytic site (closed conformation) (21). In contrast, two regions, C669–A679 and S744–W762, are in different spatial positions (open conformation) when the active site of HDAC4c is occupied by small-molecular weight inhibitors (21,23). Steric and charge repulsions between these two loops and bound bulky inhibitors can be attributed to this local rearrangement without an apparent interaction between them (21). Notably, the association of HDAC4c with the SMRT-HDAC3 complex or GSI motif-containing peptide is prevented when the open conformation is induced by inhibitor binding, mutations disrupting the structural zinc-binding domain, or redox modifications in this region (19,21,32).

The SP1- or SP2-complexed HDAC4c structures show that the bound peptides interact through their side-chains, without a noticeable structural rearrangement of side-chain atoms in the structural zinc-binding subdomain residues, compared to the apo-form structure (Figure 3C). The polar interactions of the bound peptides are also found in the catalytic subdomain, although their interactions are limited to the main-chain atoms (Figure 3D). The observed closed conformation in the complex structures was unexpected, as the bound peptides have a much bulkier volume than those of previously reported small-molecule inhibitors with an open conformation (21,23). However, this observation is consistent with the suggestion that the closed conformation in the apo-form structure may represent the biologically relevant conformation, considering that only the apo-form of HDAC4c can associate with the SMRT-HDAC3 complex or GSI motif-containing peptide (19,21,23).

Among the seven HDACs whose catalytic domain structures have been reported, HDAC4c is unique in that it has an open conformation when it binds to non-peptide molecules. In agreement with this, mutations at the ^+1^GSI^+3^ residues of two motifs and R+9 residue of the SM1 lead to a loss of its binding activity to HDAC4c/-5c (Figures 1 and 5). The class IIa HDAC-specific residues (D759, H842, and F871), which are located at the cleft to and entrance to the catalytic zinc-binding site, interact directly or indirectly with these residues of bound peptides (Figure 3D and E). Further, HDAC4c mutants of these residues (D759Y, H842L, and F871L/F871S) showed decreased binding affinities to SRD3c (20). The data obtained through structure and the structure-based mutational assays are consistent with our previous report showing that the catalytic entry site of HDAC4c/-5c, which includes most class IIa HDAC-specific residues (D759, H842, and F871), is the major binding surface of the SMRT corepressor (20) (Figure 3E).

Collectively, our results indicate that the class IIa HDAC-specific structural zinc-binding subdomain is directly involved in SRD3c binding, and the closed conformation is required for this interaction. Therefore, the closed conformation observed here can be interpreted as the biologically functional form of HDAC4 and perhaps all class IIa HDACs, which can associate with SMRT/NCoR-HDAC3 complexes.

### SRD3c binding physically blocks putative substrate access to the catalytic site of HDAC4c

The deacetylase activity of class IIa HDACs is quite low at less than 1/100-fold that of class I HDACs, as the active-site residue tyrosine in class I enzymes is substituted by histidine in class IIa HDACs (33). Interestingly, HDAC3, a class I HDAC, is inactive alone but gains its deacetylase activity upon binding to DAD of SMRT (34). Accordingly, it was proposed that the marginal catalytic activity of class IIa HDACs is increased by association of a binding factor, such as SMRT (35). Our structural studies show that two SRD3c-driven peptides are bound in the catalytic cleft without structural changes in the catalytic zinc-binding site of HDAC4c compared to in the closed conformation (Figure 3). These structural features indicate that SRD3c functions as a blocker, which may not allow the potential class IIa HDACs substrate to bind the catalytic site of HDAC4c. Therefore, it is unlikely that SMRT can induce the latent deacetylase activity of class IIa HDACs, strongly supporting the previous hypothesis that class IIa HDACs are mediator molecules that recruit the SMRT-HDAC3 complex to the transcriptional machinery, rather than acting as direct deacetylation catalysts (17).

### Molecular basis of the specific interaction of SRD3c with HDAC4c/-5c but not with class I enzymes

The structures of catalytic domains from 7 different HDACs showed identical folding with a comparable catalytic subdomain including the cleft to the active site and two conserved aromatic residues at the entrance to the deacetylase site. Nonetheless, SRD3c specifically interacts with class IIa HDACs, but not with class I enzymes (18), suggesting that a region(s) specific to class IIa enzymes is responsible for this interaction. Structural studies revealed that the structural zinc-binding subdomain is present only in class IIa HDACs and may have regulatory and structural roles (21,22,32).

**Figure 6.**
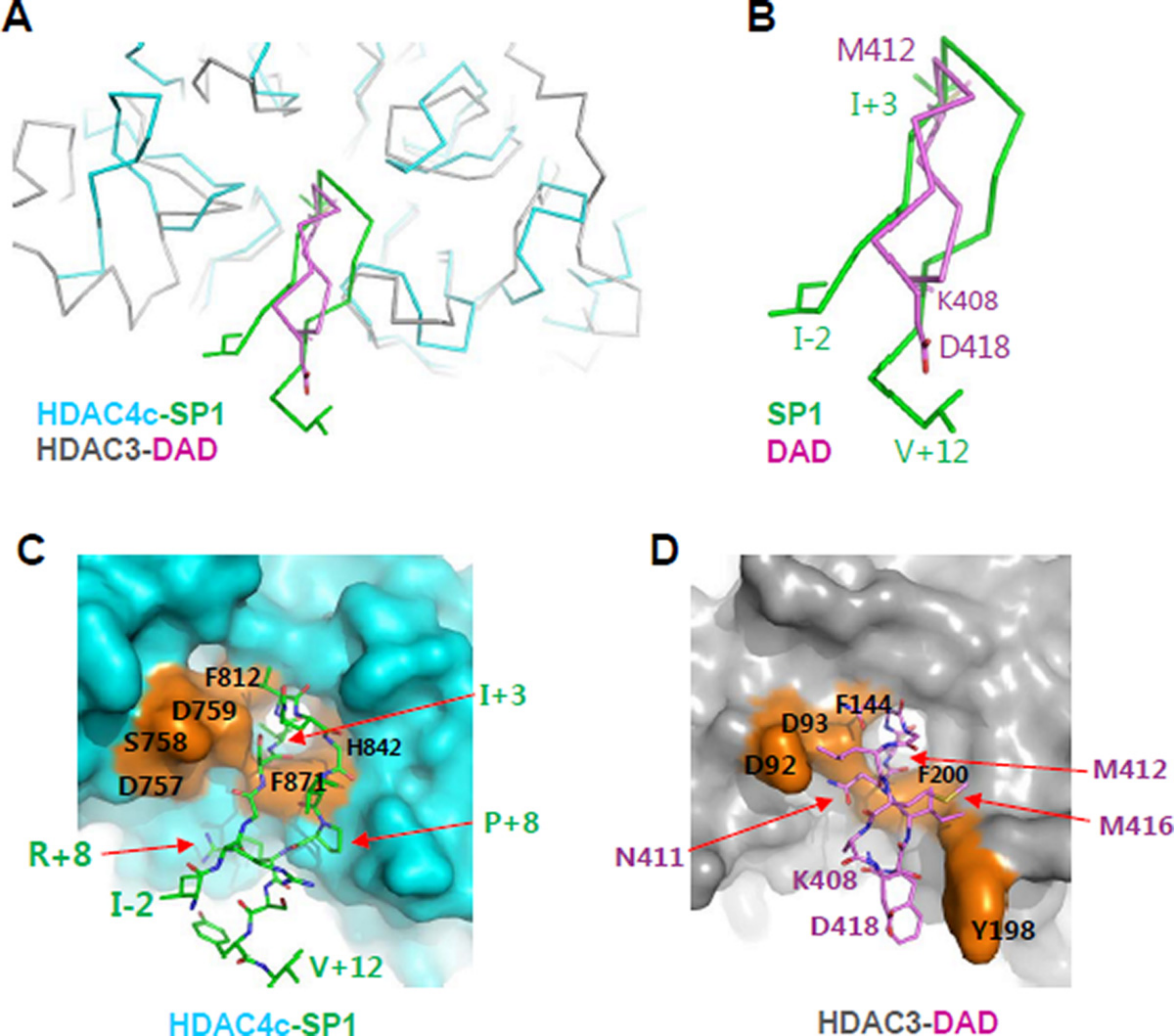
A structural comparison of the HDAC4-SP1 complex with the HDAC3-SMRT DAD complex. (**A**) Superposition of HDAC4-SP1 complex structure with HDAC3-SMRT DAD structure (PDB ID 4a69). Only the N-terminal head (K408-D418) of the SMRT DAD was displayed for clarity. Two compared structures were superposed and drawn with Cα tracings in alternative colors. Some residues were displayed with stick models. (**B**) A closed-up view of SP1 and DAD β-hairpins. Terminal residues and the key I+3 and M412 residues at the turns of two β-hairpins were displayed with sticks and labeled with different colors. (**C**) Structure of HDAC4c cleft bound with SP1 β-hairpin. HDAC4c was displayed with a surface presentation (cyan) and SP1 peptide was drawn with stick models (green). Some SP1-interacting HDAC4 residues were indicated on the surface in orange color. (**D**) Structure of HDAC3 cleft in complex with DAD β-hairpin. HDAC3 was displayed with a surface presentation (gray), and the N-terminal β-hairpin of SMRT DAD was drawn with stick models (magenta). HDAC3 residues that interact with the DAD β-hairpin were indicated on an orange-colored surface.

Our previous mutational study identified the rim surface of the HDAC4c/-5c catalytic entry site as the major SRD3c binding surface (20). In the peptide-complex structure, the β-hairpin structure in SP1 and SP2 is formed by two β-strands from the GSIxQGxPx motif and interacts specifically with two sides of the cleft wall of structural and catalytic zinc-binding subdomains (Figure 3). Interestingly, the N-terminal β-hairpin structure of SMRT DAD is in the cleft between two HDAC3 molecules, with a substrate-mimicking M412 residue sandwiched between two conserved aromatic residues at the entrance of the deacetylase site (36) (PDB ID 4a69). It is not clear whether this β-hairpin structure bound to HDAC3 was induced under the specific crystallization conditions. Nevertheless, the overall structural features of this complex appear to be significantly comparable to those observed in the β-hairpins of SP1- or SP2-HDAC4c complex (Figure 6A). However, the two β-hairpin structures from SP peptides and DAD differ in their orientations and sizes (Figure 6B–D). As described above, the ^+1^GSI^+3^ motif of the first β-strand in SP peptides and R+9 of the SP1 peptide interact intensively with class IIa HDAC-specific residues of the structural zinc-binding domain (C667, C669, C751, D759, T760, and F871), while the ^+6^GxP^+8^ motif of the second β-strand interacts using their main-chain atoms with the HDAC4c deacetylase subdomain. These critical interactions are not observed in the β-hairpin of SMRT DAD in complex with HDAC3 (36). Additionally, the bulkier SP peptides have the side-chain of the rigid proline residue (P+8) oriented to the HDAC4c deacetylase subdomain, resulting in steric collision of SP peptides with the cleft wall of the HDAC3 deacetylase domain (Figure 6C and D). Taken together, these structural features indicate that the β-hairpin of SRD3 peptides does not bind the class I HDACs, providing a molecular basis for the specific interaction of SRD3c with class IIa HDACs. Notably, our assays also showed that the SP1 and SP2 peptides did not inhibit the deacetylase activity of HDAC8, a class I HDAC (Supplementary Figure S8B).

### Suggested biological roles of class IIa HDACs

In contrast to other HDACs, class IIa HDACs have low deacetylase activity (33). However, their HDAC domains are directly involved in the transcriptional repression of several genes by forming a supra-complex with the NCoR/SMRT-HDAC3 complex (16,17). In addition to the lack of noticeable changes in the deacetylase activity of HDAC4 in the presence of SMRT (19), our complex structure suggests that their deacetylase activities are not promoted by the interaction with SMRT. Instead, our results strongly suggest that class IIa HDACs are bridge molecules between the SMRT-HDAC3 complex and transcription factors (17). Thus, it is plausible that class IIa HDACs evolved to become pseudo-enzymes, as their primary role is structural rather than catalytic.

Additional studies of the novel function of class IIa HDACs, which is independent of SMRT-HDAC3, are necessary to evaluate the physiological role of the intrinsic low HDAC activity of class IIa enzymes. Studies are also needed to determine whether the binding and inhibition of HDAC4c/-5c/-7c by SMRT/NCoR leads to changes in the acetylation pattern of target genes. Our results may be helpful for identifying and understanding the deacetylase function of class IIa enzymes, which can be inhibited by the binding of SMRT/NCoR to their active sites. Although we identified SM1 as the major binding motif of SRD to HDACs, multiple HDAC molecules may simultaneously associate with the several GSI motifs within a single chain of SMRT/NCoR. *In vivo* analysis using the SMRT/NCoR gene containing GSI motif mutation(s) or determination of the solution structure of SRD3-HDAC complex is essential for answering this fundamental question.

## DATA AVAILABILITY

Data deposition: Atomic coordinates were deposited in the Protein Data Bank, http://www.rcsb.org (accession numbers 5ZOO and 5ZOP).

## Supplementary Material

Supplementary Data
