# Supplementary material for: Structural basis of the specific interaction of SMRT corepressor with histone deacetylase 4
Source: Nucleic Acids Res. 2018 Oct 13;46(22):11776–88. doi: 10.1093/nar/gky926 (PMC6294515; doi:10.1093/nar/gky926)
Supplement: Supplementary Data [file gky926_supplemental_files.docx]

**Supplementary Information**

**Structural basis of the specific interaction of SMRT corepressor**

**with histone deacetylase 4**

Suk-Youl Park ^a,b,1^, Gwang Sik Kim ^c,1^, Hyo-Jeong Hwang ^a^, Taek-Hyun Nam ^c^, Hee-Sae Park ^c^,

Jaeyoung Song^d^, Tae-Ho Jang^d^, Young Chul Lee ^c,*^, and Jeong-Sun Kim ^a,*^

^a^ Department of Chemistry, Chonnam National University, Gwangju 61186, Republic of Korea

^b^ Pohang Accelerator Laboratory, Pohang, Gyeongbuk 37673, Republic of Korea

^c^ School of Biological Sciences and Technology, Chonnam National University, Gwangju 61186, Republic of Korea

^d^ New Drug Development Center, Daegu-Gyeongbuk Medical Innovation Foundation, Daegu 41061, Republic of Korea

^1^ These two authors are equally contributed.

^*^Correspondence should be addressed to:

Jeong-Sun Kim (Email: [jsunkim@chonnam.ac.kr](mailto:jsunkim@chonnam.ac.kr)) or

Young Chul Lee (Email: [yclee@chonnam.ac.kr](mailto:yclee@chonnam.ac.kr))

**Figure S1. SM3 motif of N-terminal region of SRD3 has a minor role in class IIa HDAC binding.** (A) Schematic diagram of SRD3T and SRD3n constructs. The first Gly of the SM1 (GSITQGIPR), SM2 (GSITQGTPL) or SM3 (GSITHGTPA) is denoted as +1. Single amino acid substitution was introduced into SM1 (I+3T), SM2 (G+1D), or SM3 (I+3T) motif of SRD3T and SRD3n and denoted as mt1, mt2, or mt3, respectively. (B) GST pull-down assay. (C, D) Yeast two-hybrid assay. Yeast strain EGY48 bearing *lexA_op_*-*LacZ* reporter was co-transformed with expression plasmids of LexA-fused HDAC4c/-5c/-7c and B42AD fusions of SRD3T/-3n/-3c constructs. Transformants were grown in synthetic minimal glucose media overnight, and then subjected to galactose induction for 5 h and liquid β-galactosidase assays. WT: wild-type. (E, F) Quantitative BiFC assay for the interaction between indicated KGN-SRD3 constructs and KGC-HAC4c/-5c proteins in HEK293 cells. WT: wild-type, E.V: empty vector, N.T: no transfection.


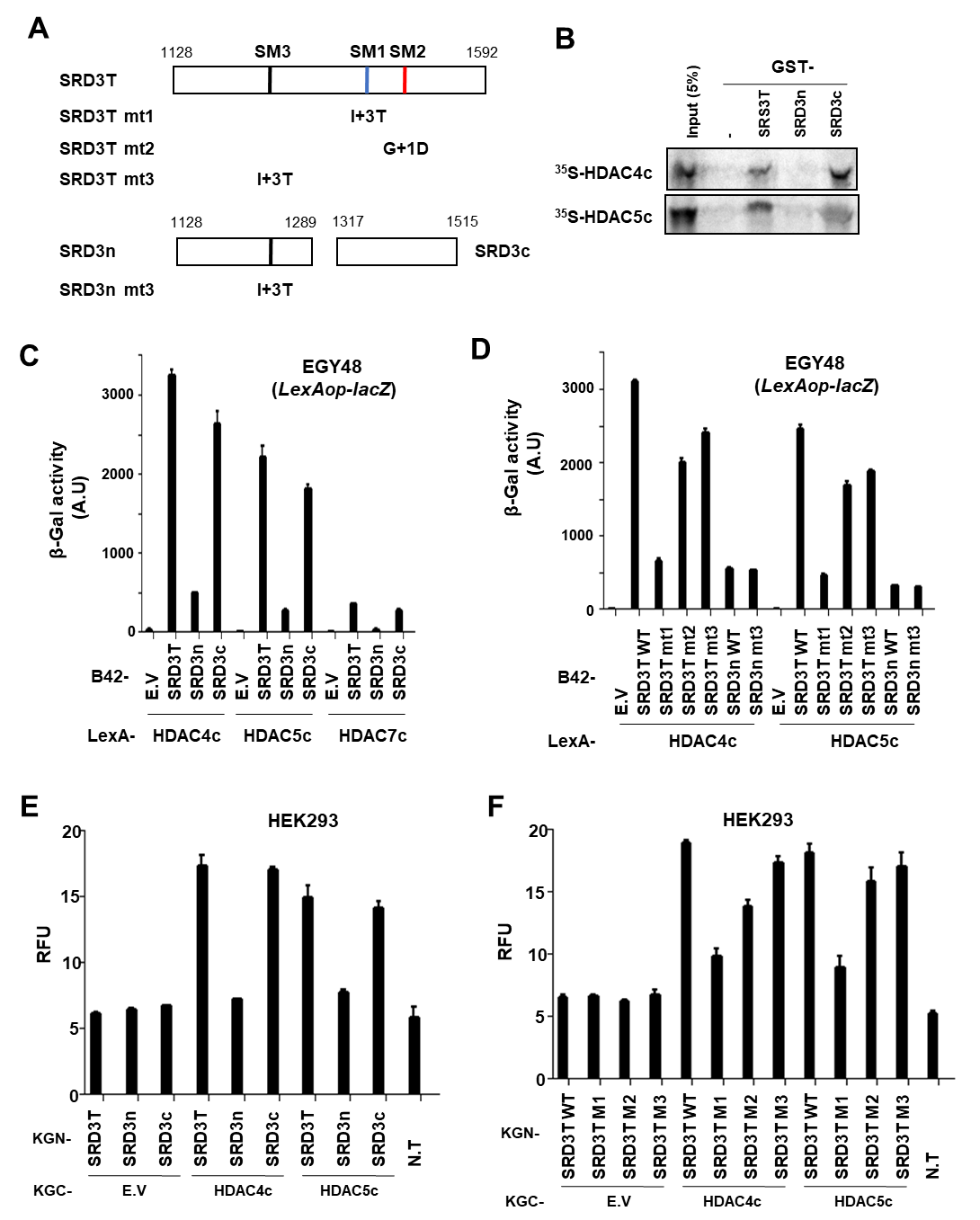


**Figure S2. Domain mapping of SRD3c region required for HDAC4c binding.** (A) Schematic presentation of SRD3c derivatives used in the mapping experiment. Full-length SRD3c (K1316–S1515), I (K1316–Q1408), I-A (K1316–G1390), I-B (K1316–H1357), I-C (H1348–Q1408) and II (P1396–S1515) fragments were used in domain mapping. (B) Protein expression levels of B42-GBD-fused SRD3c derivatives in yeast. Whole-cell lysates were prepared and subjected to immunoblot analysis using anti-GBD antibodies. (C–D) Yeast two-hybrid interaction assay. Yeast strain EGY48 bearing *lexAop*-*LacZ* reporter was co-transformed with expression plasmids for LexA-fused HDAC4c/-5c and SRD3c derivatives subcloned into the pRS324UBG vector. Transformants were grown in synthetic minimal glucose media and subjected to liquid β-galactosidase assays.


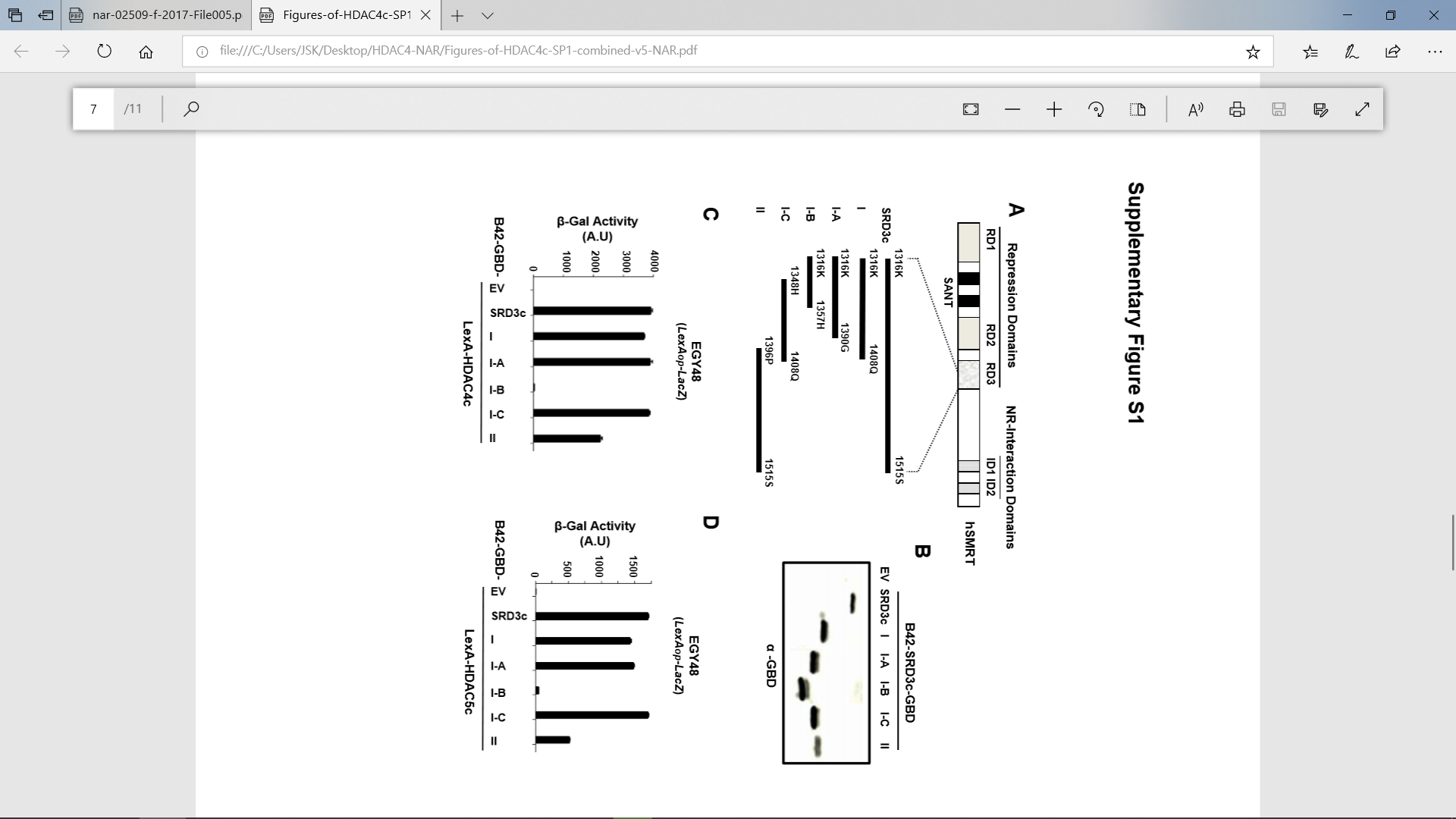


**Figure S3. Defective interactions of isolated SM1 mutants with HDAC4c/-5c.** (A) Positions and amino acid changes of isolated SM1 mutant alleles by OPTHiS screening. (B) Defective interactions of indicated LexA-SM1F mutants with B42-GBD-HDAC4c/-5c in yeast two-hybrid assays. (C) *In vitro* GST-pull down assay. *In vitro* synthesized HDAC4c/-5c were tested for their binding to GST or GST-fused SM1F derivatives. (D) Quantitative BiFC assay for the interaction between indicated KGN-SM1F derivatives and KGC-HDAC4c/-5c proteins in HEK293 cells. E.V: empty vector, N.T: no transfection. (E) Confocal laser-scanning image for BiFC interaction assay between indicated KGN-SM1F derivatives and KGC-HDAC4c. *Magnification*: 60 X.


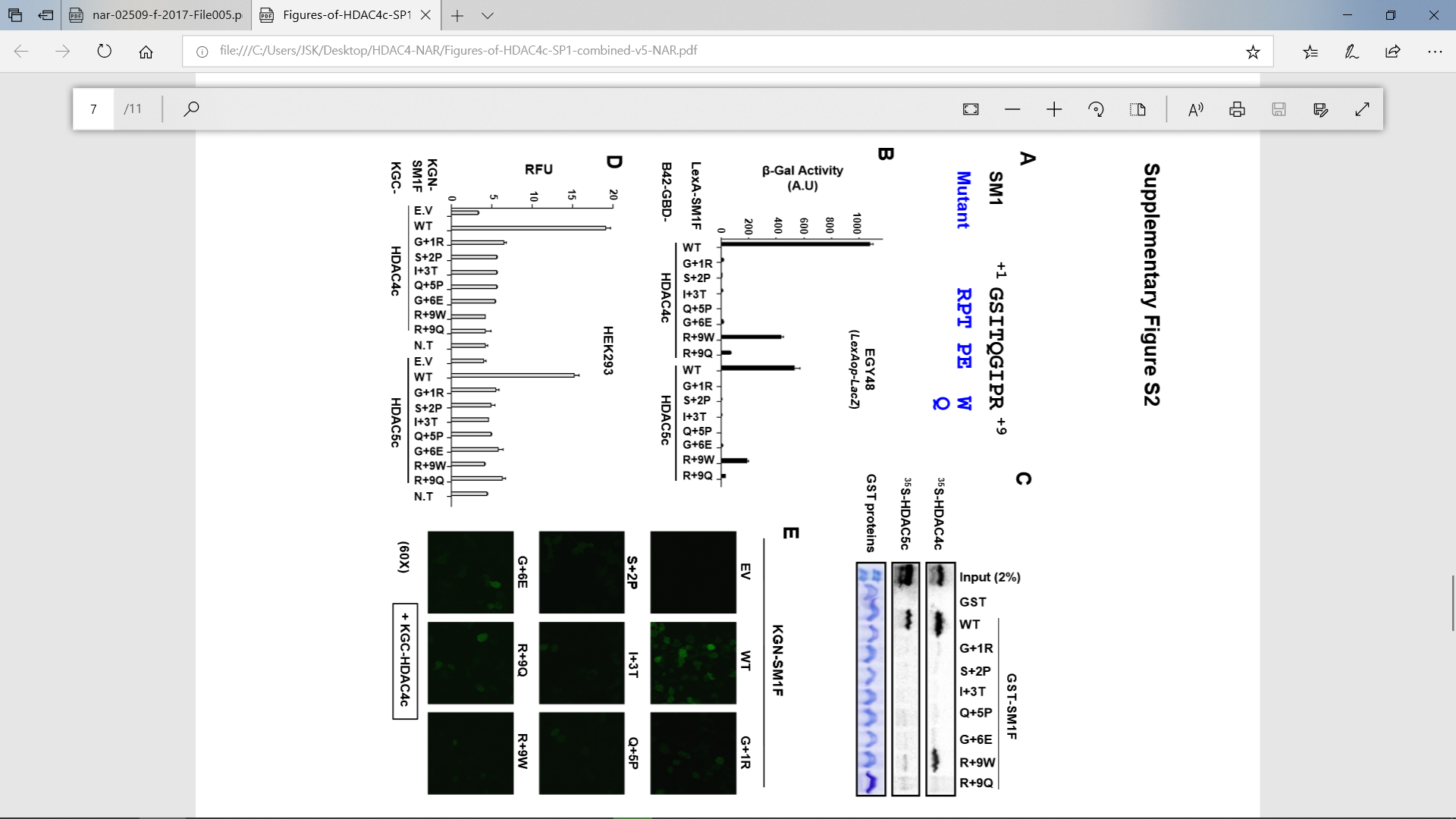


**Figure S4. Defective interactions of isolated SM2 mutants with HDAC4c/-5c.** (A) The positions and amino acid changes of isolated SM2 mutant alleles by OPTHiS screening. (B) Defective interactions of indicated LexA-SM2F mutants with B42-GBD-HDAC4c/-5c in yeast two-hybrid assays. (C) *In vitro* GST-pull down assay. *In vitro* synthesized HDAC4c/-5c were tested for their binding to GST or GST-fused SM2F derivatives. (D) Quantitative BiFC assay for the interaction between indicated KGN-SM2F mutants and KGC-HDAC4c/-5c proteins in HEK293 cells. E.V: empty vector, N.T: no transfection.


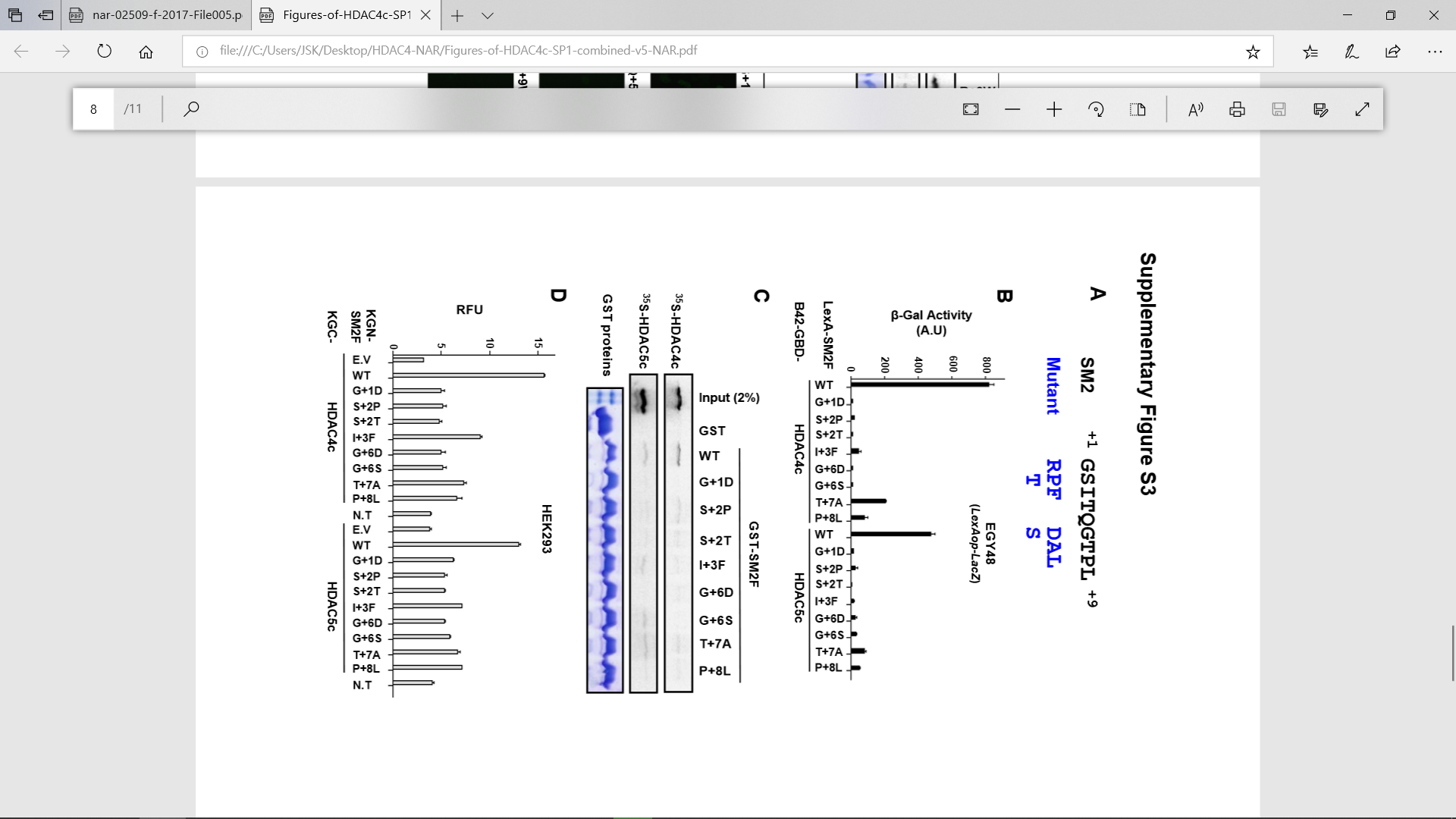


**Figure S5. Yeast two-hybrid interactions of HDAC7c with SRD3c and SM1F/-2F mutants.** Yeast two-hybrid interactions of HDAC7c with SRD3c derivatives (wild-type, mt1, mt2, or dmt) (A), SM1F/-2F mutants obtained by OPTHiS screening (B, C), and synthetic mimic mutants of SM1F/-2F (D). Yeast strain EGY48 bearing *LexA_op_*-*LacZ* reporter was co-transformed with expression plasmids for indicated LexA-fused constructs and B42AD-fused HDAC7c. Transformants were grown in synthetic minimal glucose media overnight, followed by galactose induction for 5h, and subjected to liquid β-galactosidase assay. WT: wild-type.


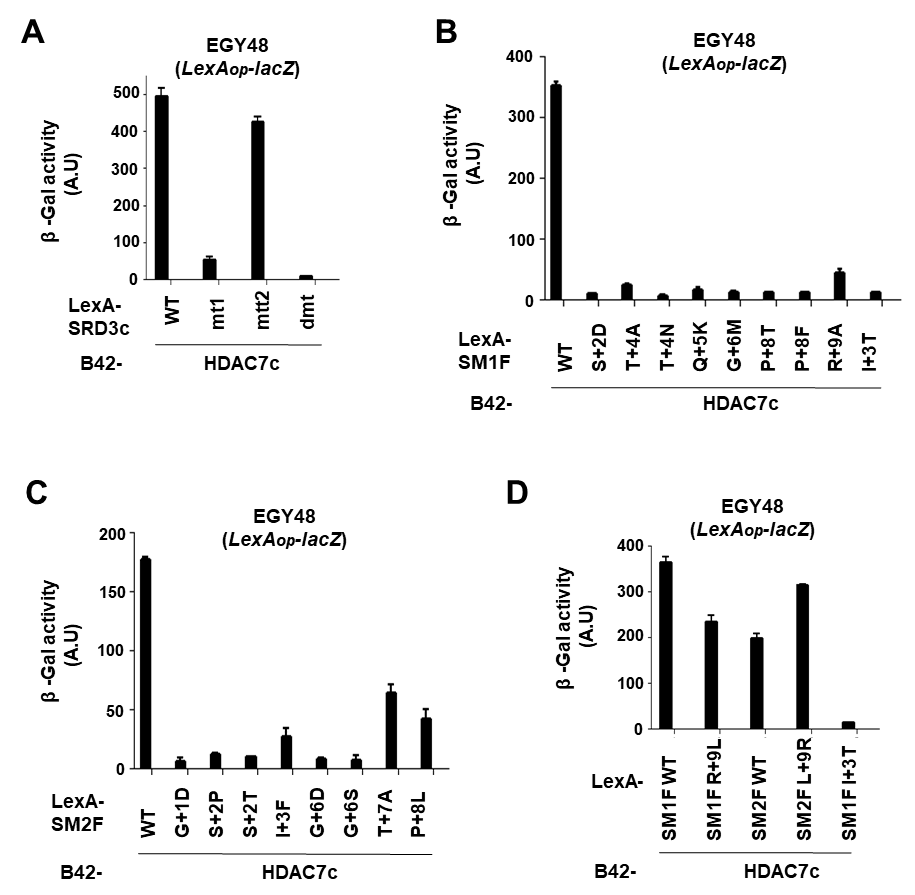


**Figure S6. Independent interactions of HDAC4c/-5c with SM1 and SM2 fragment of SRD3c.** (A) Schematic presentation of SM1F (E1346–L1385) and SM2F (H1443–R1482) fragments containing SM1 and SM2 motifs, respectively. (B) Yeast two-hybrid assay. LexA-fused SM1F or SM2F were tested for binding to B42-GBD-HDAC4c/-5c in EGY48 strain. (C) *In vitro* GST pull-down assay. *In vitro* synthesized HDAC4c/-5c were examined for their binding to GST or GST-fused SM1F/-2F proteins. (D) Quantitative BiFC assay for the interaction of KGN-SM1F or -SM2F with KGC-HAC4c/-5c proteins in HEK293 cells. E.V: empty vector, N.T: no transfection. (E) Confocal laser scanning image for BiFC interaction assay of KGN-SM1F or -SM2F proteins with KGC-HDAC4c/-5c. *Magnification*: 60 X.


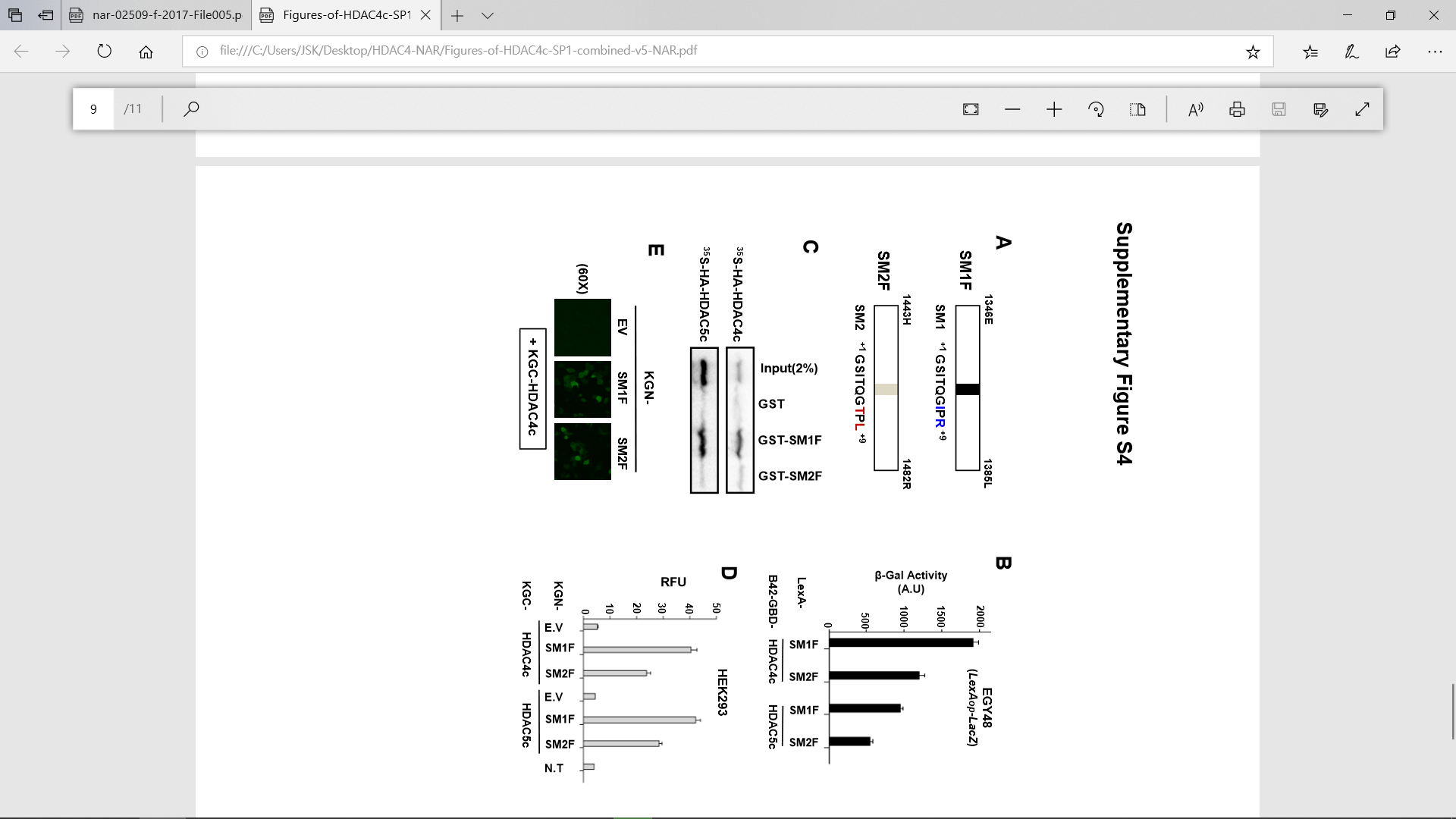


**Figure S7. Isothermal titration calorimetry (ITC) curves for the binding of SP1 (A) and SP2 (B) to the HDAC4c H976Y.** The top panels show the raw ITC data generated by titration of HDAC4c by 25 injections of SP1 and SP2. The area under each peak was integrated and plotted against the injection of SP1 and SP2 to HDAC4cd, respectively. The solid smooth lines represent the best fit of the experimental data according to a single-site binding model. The resultant thermodynamic parameters are summarized in Table 2.


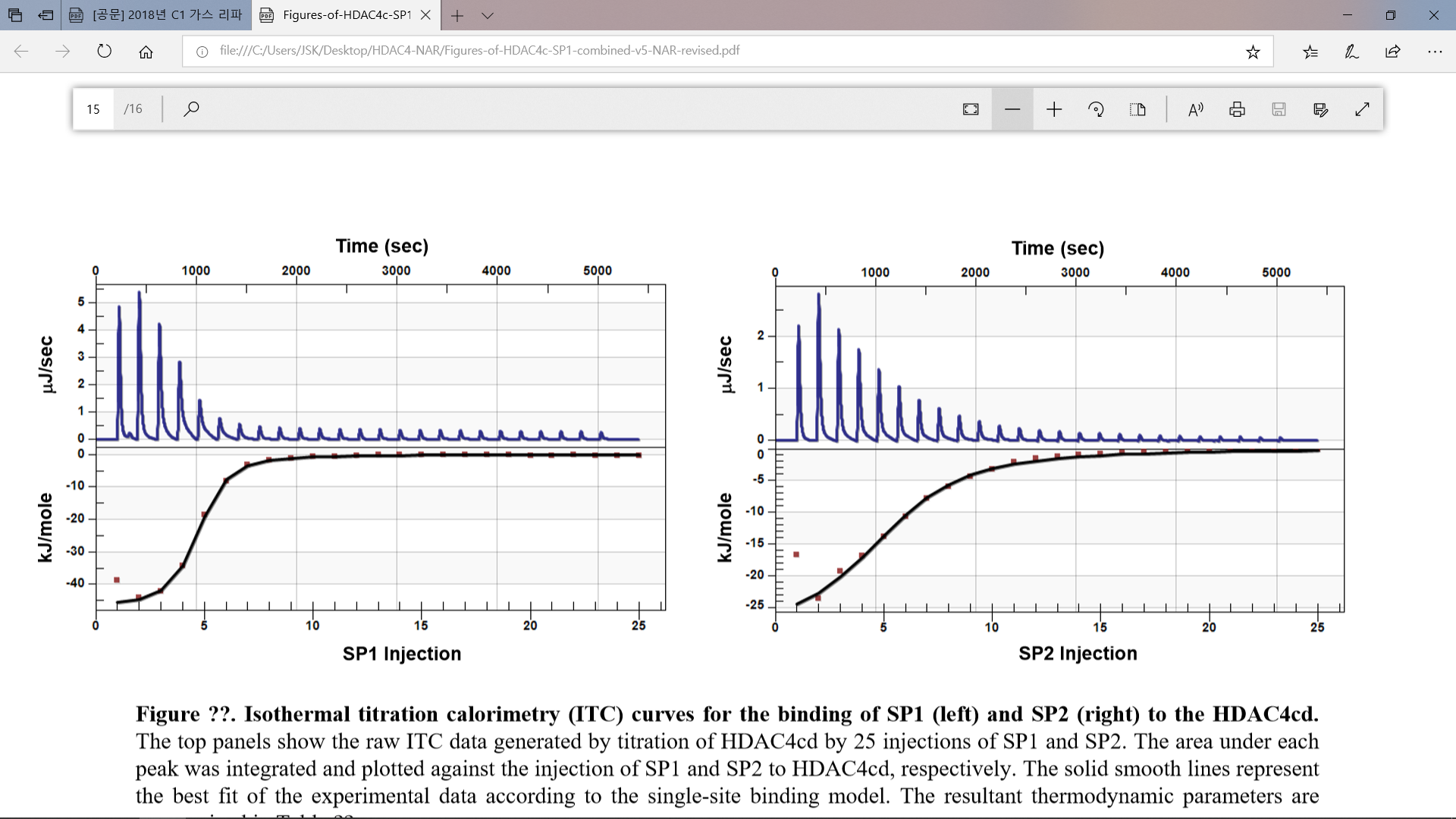

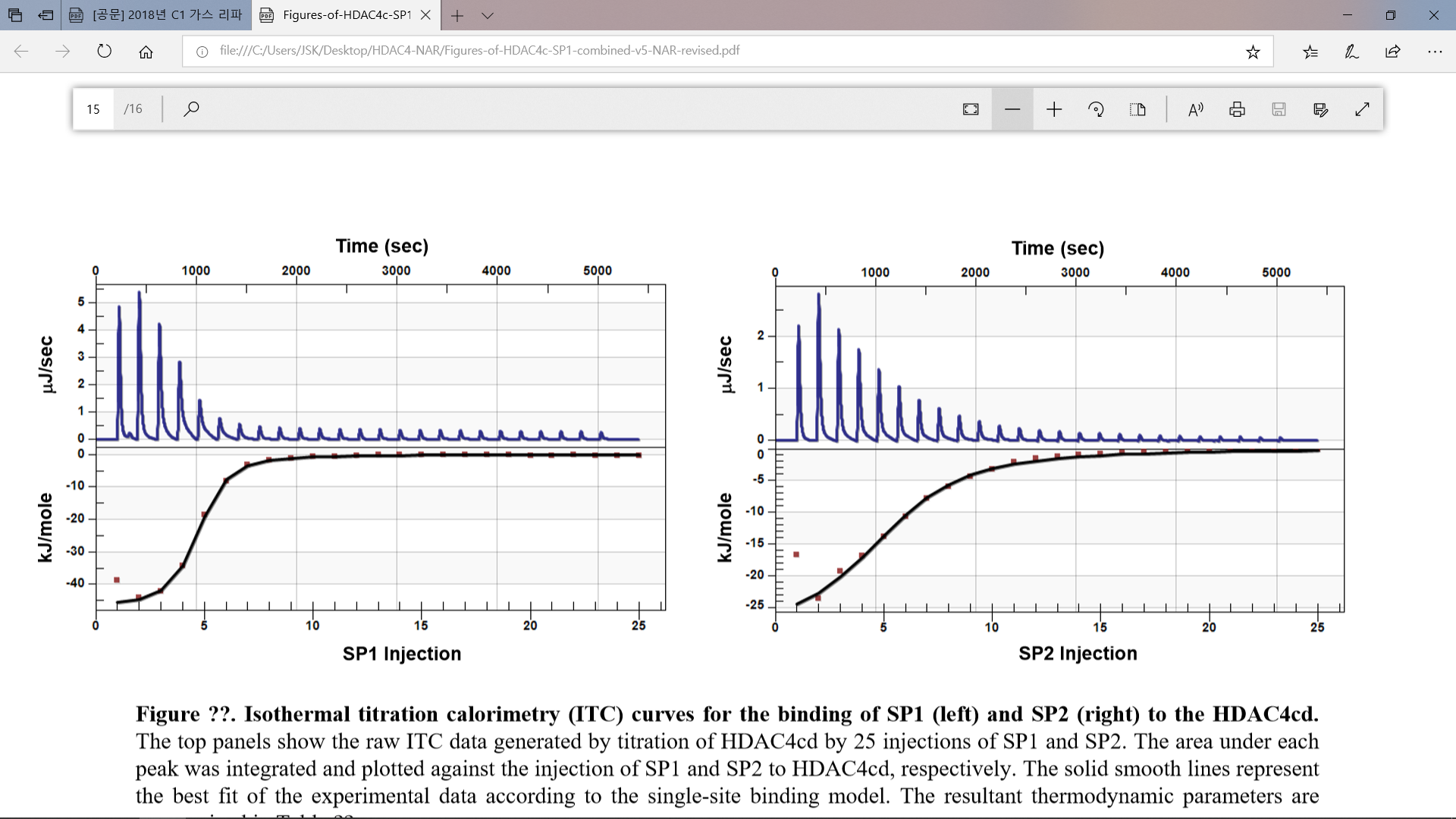


**A**

**B**

**Figure S8. Synthetic peptides SP1 and SP2 inhibit *in vitro* HDAC activity of HDAC4c H976Y, but not of HDAC8 activity.** *In vitro* HDAC activities of HDAC976Y (A) and GST-HDAC8 (B) were measured using fluorescent-coupled Lys acetamide as substrate in the presence of increasing concentrations of SP1 or SP2 (4 nM ~ 62.5 μM). The fluorescence signal generated by HDAC activity was quantitated with a fluorescence spectrometer. RFU: Relative Fluorescence Unit.


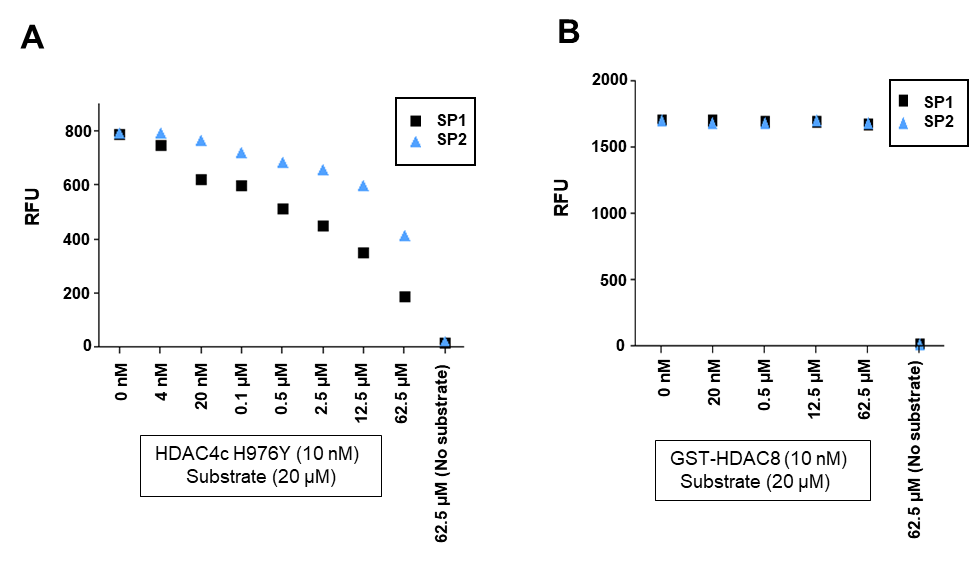


**Figure S9. Electron density maps for the SP-complexed HDAC4c.** Residues at the structural zinc-binding site (A) and the catalytic site (B) are displayed with line models in the 2*Fo-Fc* electron density map contoured at 1σ. The Zn ions (Zn) and water molecules are shown as black and red spheres, respectively. The SP1 (C) and SP2 (D) peptides and nearby HDAC4c residues are displayed with stick models in the refined 2*Fo-Fc* electron density map (orange) contoured at 1σ.


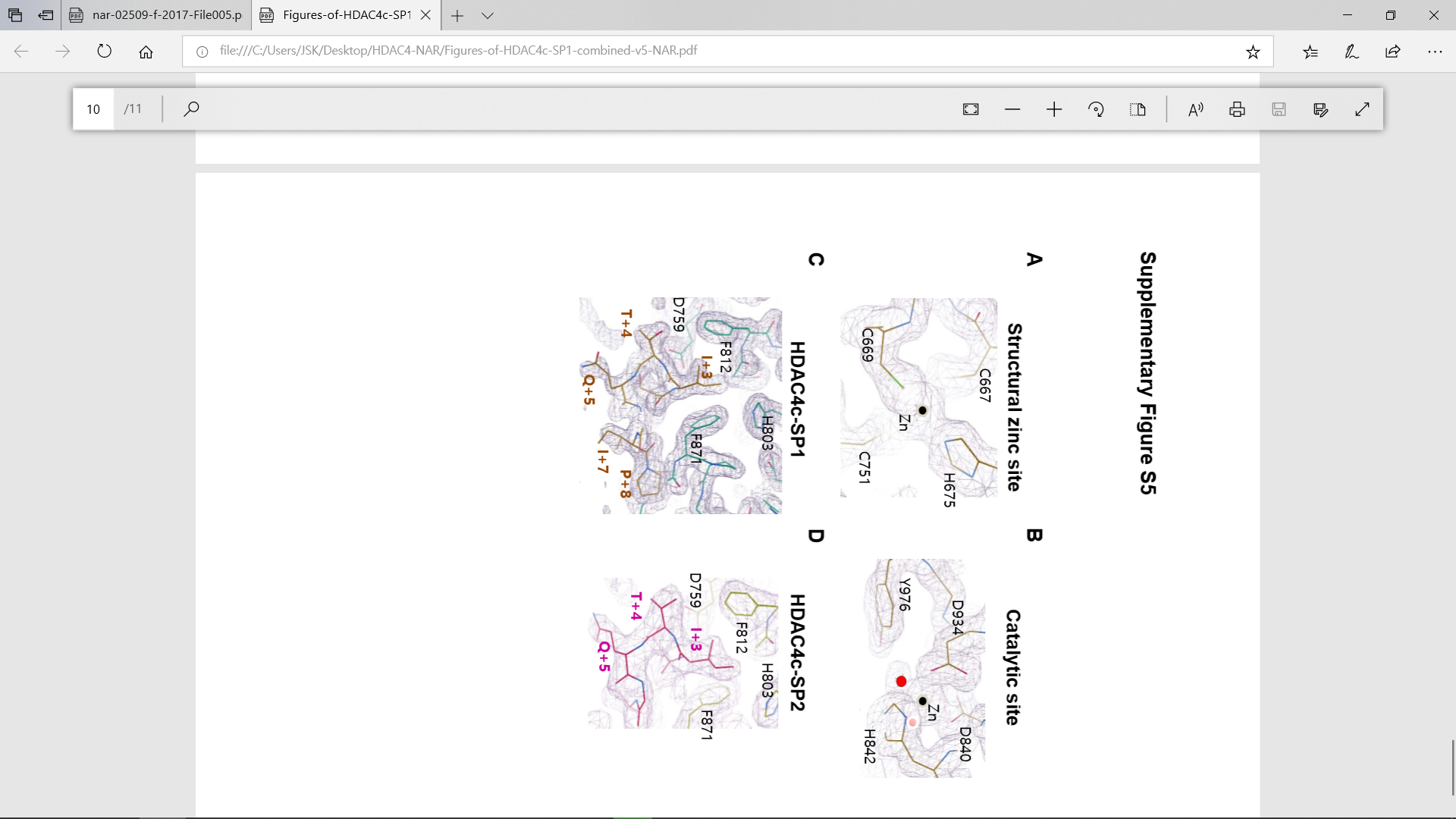
**Supplementary Table**

Table S1. Catalytic zinc ion

| Metal | Protein | Water | Distance (Å) |
| --- | --- | --- | --- |
| Zn | OD1 (D840) |  | 2.04 |
|  | OD2 (D840) |  | 2.58 |
|  | OD2 (D934) |  | 2.00 |
|  | ND1 (H842) |  | 2.09 |
|  |  | w1  w2 | 2.16  2.09 |
|  | OH (Y976) | w1 | 2.39 |
|  | NE2 (H802) | w2 | 2.69 |
|  |  | w2-w3  w1-w3 | 2.64  2.69 |
|  | OH (Y976) | w3 | 3.21 |

Table S2. Polar interactions between the SP1 peptide and protein atoms

| SP1-peptide | | Protein | Water | Distance (Å) |
| --- | --- | --- | --- | --- |
| G1361  (G+1) | C=O |  | w6 | 3.17 |
|  |  | OG (S758) | w6 | 2.82 |
| S1362  (S+2) | OG |  | w5 | 2.53 |
|  |  | OG (S758) | w5  w6 | 2.66  2.70 |
| I1363  (I+3) | NH (peptidyl) | OD2 (D759) |  | 2.89 |
|  | C=O |  | w4 | 2.68 |
|  |  | NE2 (H842) | w4 | 2.82 |
|  |  | O=C (P942, peptidyl oxygen) | w4 | 2.69 |
| T1364  (T+4) | OG | OD1 (D759, peptidyl oxygen) |  | 2.96 |
| R1369  (R+9) | NH (peptidyl) | O=C (W131, peptidyl oxygen) |  |  |
|  | NH1 |  | w8 | 2.78 |
|  |  |  | w6-w7 | 2.71 |
|  |  |  | w7-w8 | 2.73 |
|  |  | OD2 (D759) | w7 | 2.78 |

**Supplementary Methods**

**Immunoblot analysis using yeast whole cell extracts**

Yeast whole cell extracts were prepared as described previously (25). Each protein sample (approximately 40 μg) was separated by 12% SDS-PAGE and transferred to Hybond-ECL nitrocellulose membranes (GE Healthcare, Little Chalfont, UK). Membrane was probed with anti-GBD (sc-510; Santa Cruz Biotechnology, Dallas, TX, USA) to detect the expression levels of B42-GBD-fused SRD3c protein derivatives. Immunoblots were developed using the Optiblot ECL ultra detection kit (Abcam, Cambridge, UK) and images were acquired using an HP imaging system.

***In vitro* HDAC assay**

The activity of HDAC was measured using an HDAC assay kit (BioVision, Inc., Milpitas, CA, USA) according to the manufacturer's protocol. Briefly, synthetic peptide (SP1 or SP2) and purified recombinant HDACs were pre-incubated 15 min at 37°C in HDAC assay buffer, followed by the addition of 20 μM fluorogenic substrate [R-H-K-K(Ac)-AMC]. Reaction mixtures were incubated for 30 min at 37°C and the fluorescent signals (excitation wavelength: 360 nm, emission wavelength: 450 nm) were measured using a fluorescence plate reader (Molecular Devices, San Jose, CA, USA).

**Plasmids**

To construct the expression vector for GST-fused HDAC8, human HDAC8 region (M1–V377) was amplified by PCR using pcDNA3-HDAC8-Flag (Addgene, donated by Eric Verdin) as a template and then inserted into the EcoRI/XhoI sites of the pGEX4T-1 vector. To construct expression vectors for SRD3T (R1128–K1592 of hSMRT) and SRD3n (R1128–E1289), each PCR-amplified DNA fragment was inserted into the EcoRI/XhoI sites of the pB42, pcDNA3-HA, or pGEX4T-1 vectors, respectively. To introduce point mutation(s) into the pB42-SRD3T (mt1, mt2, and mt3), or -RD3N mt3, site-directed mutagenesis was performed using Quickchange II site-directed mutagenesis kit according to the manufacturer’s instruction (Agilent Technologies, Santa Clara, CA, USA). To prepare KGN-MC-SRD3T or -SRD3n, the KpnI/XhoI fragment from pcDNA3-HA-SRD3T or -SRD3n was ligated into the corresponding sites of the KGN-MC vector. The mutant derivatives of KGN-MC-SRD3T were prepared by site-directed mutagenesis. To construct the expression vectors for B42-fused HDAC7c, the mouse HDAC7 catalytic region (L460–P939) was amplified by PCR using specific oligomers and inserted into the EcoRI/XhoI sites of the pB42 vector. All DNA sequences were confirmed by DNA sequencing.
